# Bioactive glycans in a microbiome-directed food for children with malnutrition

**DOI:** 10.1038/s41586-023-06838-3

**Published:** 2023-12-13

**Authors:** Matthew C. Hibberd, Daniel M. Webber, Dmitry A. Rodionov, Suzanne Henrissat, Robert Y. Chen, Cyrus Zhou, Hannah M. Lynn, Yi Wang, Hao-Wei Chang, Evan M. Lee, Janaki Lelwala-Guruge, Marat D. Kazanov, Aleksandr A. Arzamasov, Semen A. Leyn, Vincent Lombard, Nicolas Terrapon, Bernard Henrissat, Juan J. Castillo, Garret Couture, Nikita P. Bacalzo, Ye Chen, Carlito B. Lebrilla, Ishita Mostafa, Subhasish Das, Mustafa Mahfuz, Michael J. Barratt, Andrei L. Osterman, Tahmeed Ahmed, Jeffrey I. Gordon

**Affiliations:** 1 Edison Family Center for Genome Sciences and Systems Biology, Washington University School of Medicine, St Louis, MO USA; 2 Center for Gut Microbiome and Nutrition Research, Washington University School of Medicine, St Louis, MO USA; 3 Department of Pathology and Immunology, Washington University School of Medicine, St Louis, MO USA; 4 Infectious and Inflammatory Disease Center, Sanford Burnham Prebys Medical Discovery Institute, La Jolla, CA USA; 5 Architecture et Fonction des Macromolécules Biologiques, CNRS, Aix-Marseille University, Marseille, France; 6 Faculty of Engineering and Natural Sciences, Sabanci University, Istanbul, Turkey; 7 Department of Biotechnology and Biomedicine (DTU Bioengineering), Technical University of Denmark, Lyngby, Denmark; 8 Department of Biological Sciences, King Abdulaziz University, Jeddah, Saudi Arabia; 9 Department of Chemistry, University of California, Davis, Davis, CA USA; 10 International Centre for Diarrhoeal Disease Research, Bangladesh (icddr,b), Dhaka, Bangladesh

**Keywords:** Microbiome, Metagenomics

## Abstract

Evidence is accumulating that perturbed postnatal development of the gut microbiome contributes to childhood malnutrition^1–4^. Here we analyse biospecimens from a randomized, controlled trial of a microbiome-directed complementary food (MDCF-2) that produced superior rates of weight gain compared with a calorically more dense conventional ready-to-use supplementary food in 12–18-month-old Bangladeshi children with moderate acute malnutrition^4^. We reconstructed 1,000 bacterial genomes (metagenome-assembled genomes (MAGs)) from the faecal microbiomes of trial participants, identified 75 MAGs of which the abundances were positively associated with ponderal growth (change in weight-for-length *Z* score (WLZ)), characterized changes in MAG gene expression as a function of treatment type and WLZ response, and quantified carbohydrate structures in MDCF-2 and faeces. The results reveal that two *Prevotella copri* MAGs that are positively associated with WLZ are the principal contributors to MDCF-2-induced expression of metabolic pathways involved in utilizing the component glycans of MDCF-2. The predicted specificities of carbohydrate-active enzymes expressed by their polysaccharide-utilization loci are correlated with (1) the in vitro growth of Bangladeshi *P. copri* strains, possessing varying degrees of polysaccharide-utilization loci and genomic conservation with these MAGs, in defined medium containing different purified glycans representative of those in MDCF-2, and (2) the levels of faecal carbohydrate structures in the trial participants. These associations suggest that identifying bioactive glycan structures in MDCFs metabolized by growth-associated bacterial taxa will help to guide recommendations about their use in children with acute malnutrition and enable the development of additional formulations.

## Abstract (web summary)

Two *Prevotella copri* metagenome-assembled genomes that are positively associated with ponderal growth are the principal contributors to MDCF-2-induced expression of metabolic pathways involved in utilizing the component glycans of MDCF-2—a microbiome-directed complementary food.

## Main

The global health challenge of childhood undernutrition is considerable; in 2020, an estimated 149 million children under the age of 5 years had stunted growth (low height for age) while 45 million exhibited wasting (low WLZ)^5^. Undernutrition and its long-term sequelae are the leading causes of morbidity and mortality in individuals of this age range. Sequelae include persistent impairments in linear growth, immune and metabolic functions, and neurodevelopment—all of which have proven to be largely resistant to current interventions^6^. Although food insecurity is not the sole driver of undernutrition^7^, the profound disruption of economies and food systems by the COVID-19 pandemic has greatly exacerbated this global health challenge^8^.

Studies of healthy members of birth cohorts living in several countries have identified shared features of gut microbial community assembly—a process that is largely completed by the end of the second postnatal year^9,10^. Children with moderate (MAM) or severe (SAM) acute malnutrition have impaired ponderal growth (wasting). Their microbial community development is perturbed, resulting in microbiota configurations that resemble those of chronologically younger children^9^. The metabolic maturation of children with malnutrition is also compromised compared with their healthy peers^11^. Colonization of gnotobiotic mice with faecal microbiota samples collected from healthy children or from chronologically age-matched children with acute malnutrition revealed that microbial communities from the latter transmitted impaired weight-gain and altered bone-growth phenotypes, and produced immune and metabolic abnormalities^1,2,12^.

We used gnotobiotic mouse and piglet models to design MDCF formulations for repairing the microbial communities of children with MAM. MAM is defined as having a WLZ score that is 2–3 s.d. below the median of a multinational cohort of age-matched healthy children. In a 3-month randomized controlled feeding study of 12–18-month-old Bangladeshi children with MAM, we demonstrated that a lead formulation (MDCF-2) produced a significant improvement in the rate of weight gain (*β*-WLZ) compared with a conventional ready-to-use supplementary food (RUSF) that was not designed to alter the gut microbiota^4^. The superior effect of MDCF-2 on *β*-WLZ occurred even though its caloric density is 15% lower than RUSF. Plasma proteomic analyses revealed 70 proteins of which the levels had statistically significant positive correlations with the change in WLZ, including mediators of musculoskeletal growth and neurodevelopment. These proteins were increased to a significantly greater degree in MDCF-2-treated children compared with in those receiving RUSF. The levels of several proteins involved in immunoinflammatory processes were negatively correlated with WLZ and significantly reduced by MDCF-2 treatment^4^. Sequencing PCR amplicons generated from bacterial 16S rRNA genes present in faecal biospecimens revealed 23 bacterial taxa that were significantly associated with WLZ; 21 were positively associated, whereas two were negatively associated. The abundances of the positively associated taxa increased to a significantly greater degree after treatment with MDCF-2 compared with RUSF^4^.

Here we reconstruct the genomes of bacteria present in the gut communities of the participants in the completed trial, identify metabolic pathways that are differentially expressed in response to MDCF-2 in MAGs that are positively associated with WLZ and determine how their differential expression relates to the processing of components of MDCF-2 and ponderal growth responses. The results highlight the marked strain specificity of microbiome responses and point to two *P. copri* strains as key mediators of MDCF-2 glycan metabolism and host ponderal growth responses.

## Bacteria associated with growth

**Fig. 1:**
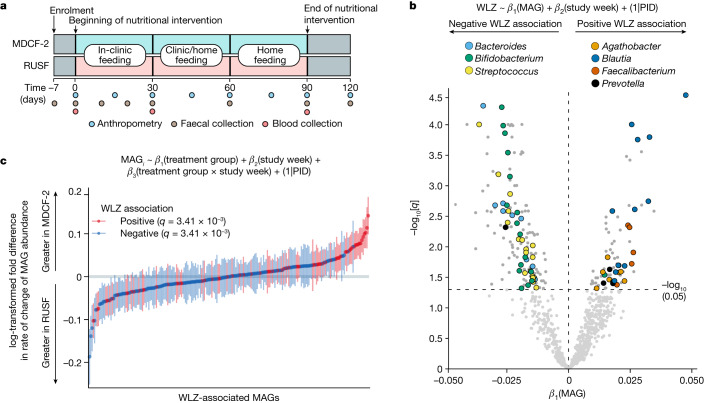
Identification of WLZ-associated MAGs. **a**, The human study design. **b**, The results of linear mixed-effects modelling of the relationship (indicated by a ~) between MAG abundance and WLZ scores for all of the trial participants, irrespective of treatment. Bacterial genera that are prevalent in the list of MAGs significantly associated with WLZ are coloured by their taxonomic classification. PID, participant identifier. **c**, The results of GSEA of WLZ-associated MAGs ranked according to the magnitude of the difference in their rate of change in abundance over time in response to MDCF-2 versus RUSF treatment. The plotted values indicate the mean ± s.e.m. log_2_-transformed fold change in the *β*_3_(treatment group × study week) coefficient for 589 biologically independent samples across the *n* = 59 participants assigned to each of the two treatment groups. The statistical significance of enrichment (*q* value, GSEA) of MAGs that are positively or negatively associated with WLZ is shown.

A summary of the design of the randomized, controlled feeding study of children with MAM, aged 15.4 ± 2.0 months (mean ± s.d.) at enrolment is shown in Fig. 1a. These children lived in an urban area with high levels of poverty (Mirpur) located in Dhaka, Bangladesh. The 3-month intervention involved twice-daily dietary supplementation with either MDCF-2 or RUSF (two 25 g servings, providing around 220–250 kcal per day)^4^. A total of 59 children in each treatment group completed the intervention and a 1-month follow-up; faecal samples were collected every 10 days during the first month and every 4 weeks thereafter. There were no statistically significant differences in the amount of nutritional supplement consumed between children receiving MDCF-2 versus RUSF, no differences in the proportion of children who satisfied World Health Organization requirements for minimum meal frequency or minimum acceptable diet, and no differences in the amount of breast milk consumed between the two treatment groups^4^.

**Extended Data Fig. 1:**
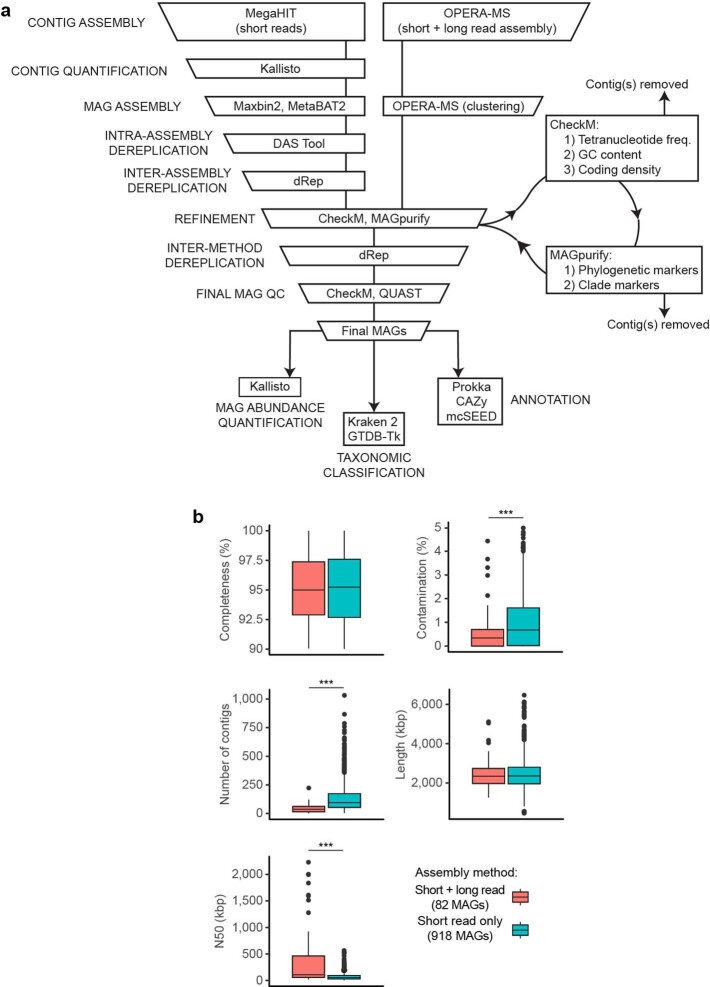
Bioinformatic workflow for MAG assembly, refinement, and quantitation. **(a)** Pipeline for MAG assembly from short-read only or short-read plus long-read shotgun sequencing data. Steps are indicated alongside the boxes, while the bioinformatic tools employed to accomplish each step are described within each box. **(b)** Comparison of MAG assembly summary statistics derived from CheckM (completeness, contamination) or Quast (number, length and N50 of contigs) for n = 82 high-quality MAGs obtained from short- plus long-read hybrid assemblies versus n = 918 high-quality MAGs from short-read only assembly methods. MAGs were assembled from shotgun sequencing data obtained from n = 942 biologically independent faecal samples as described in *Methods*. Boxplots show the median, first and third quartiles; whiskers extend to the largest value no further than 1.5 × the interquartile range. ***, *P* < 0.001 (Wilcoxon test, two-sided).

**Extended Data Fig. 2:**
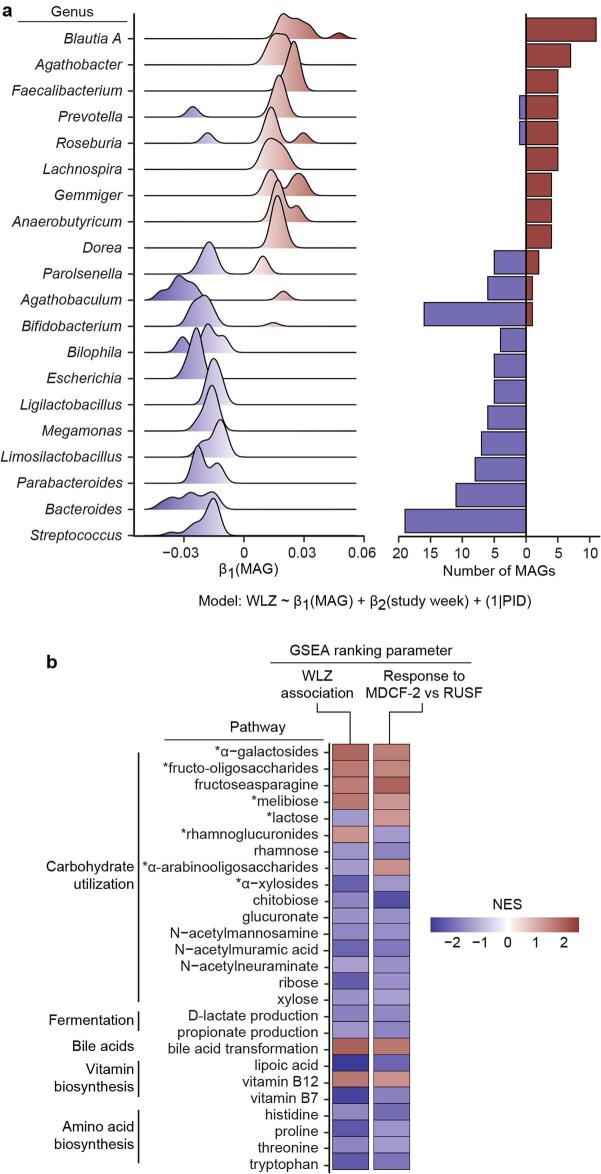
Taxonomy and functional characteristics of WLZ-associated MAGs. **(a)** Left subpanel, density plot showing WLZ-associated MAGs tabulated based on their genus-level classification. β_1_ refers to the coefficient in the linear mixed effects model presented at the bottom of the figure. Genera containing >3 significantly WLZ-associated MAGs are shown. Right subpanel, number of statistically significant WLZ-associated MAGs assigned to each genus depicted in the left subpanel. **(b)** Enrichment of metabolic pathways in WLZ- and treatment-associated MAGs. MAGs were ranked by their WLZ association (negative to positive) or treatment association (RUSF-associated to MDCF-2-associated) and GSEA was employed to determine overrepresentation of pathways in MAGs at the extremes of each ranked list. The results (Normalized Enrichment Score, NES) only include pathways that display a statistically significant enrichment (q<0.05, GSEA) in both the WLZ-associated MAG and treatment-associated MAG analyses. For carbohydrate utilization pathways, disaccharides and oligosaccharides are indicated with an asterisk.

To reconstruct the genomes of bacterial taxa present in the gut microbiomes of the study participants, we isolated DNA from all of the faecal samples (*n* = 942; 7–8 samples per participant) and performed short-read shotgun sequencing. DNA recovered from faecal biospecimens collected at *t* = 0 and 3 months from the subset of participants comprising the upper quartile of the ponderal growth response to MDCF-2 (*n* = 15)^4^ were analysed using additional long-read sequencing. We assembled pooled shotgun sequencing data from each participant’s faecal samples (short-read only, or short plus long reads when available) and aggregated contigs into MAGs (Extended Data Fig. 1, Methods and Supplementary Discussion). The resulting set of 1,000 high-quality MAGs (defined as ≥90% complete and ≤5% contaminated based on marker gene analysis; Supplementary Table 1a) represented 65.6 ± 8.0% and 66.2 ± 7.9% of all quality-controlled, paired-end shotgun reads generated from all 942 faecal DNA samples analysed in the MDCF-2 and RUSF treatment groups, respectively (2.3 ± 1.4 × 10^7^ 150-nucleotide paired-end reads per sample (mean ± s.d.); Supplementary Table 2a). Taxonomy was assigned to MAGs^13^ (Supplementary Table 1a). Abundances were calculated for each MAG in the 707 faecal samples that spanned the beginning of treatment to the timepoint at 1-month after intervention and for which matching anthropometric measurements from children had been collected. A total of 837 MAGs satisfied our abundance and prevalence thresholds (Methods and Supplementary Table 2b). We then used linear mixed-effects models to identify 222 MAGs of which the abundances were significantly associated with WLZ (*β*_1_(MAG), false-discovery-rate-adjusted *P* (*q*) < 0.05; Fig. 1b) over the 90-day course of the intervention and 30-day follow-up (the 75 positively associated and 147 negatively associated MAGs are shown in Supplementary Table 3). MAGs that were significantly positively associated with WLZ were predominantly members of the genera *Agathobacter*, *Blautia*, *Faecalibacterium* and *Prevotella*, whereas members of *Bacteroides*, *Bifidobacterium* and *Streptococcus* were prevalent among MAGs that were negatively associated with WLZ (Fig. 1b, Extended Data Fig. 2a and Supplementary Table 3).

Changes in MAG abundances were subsequently modelled as a function of treatment group, study week and the interaction between treatment group and study week, controlling for repeated measurements taken from the same individual (Fig. 1c (equation) and Methods). The ‘treatment group × study week’ interaction coefficient in the equation describes the difference in the rate of change in abundance of a given MAG (Fig. 1c). Restricting this analysis to the time of initiation of treatment did not reveal any statistically significant differences in MAG abundances between the two groups (*q* > 0.05, one linear model per MAG; Supplementary Table 3b). Expanding the analysis to include all timepoints from initiation to the end of treatment revealed that, although no individual MAG abundances were significantly associated with MDCF-2 or RUSF consumption, MAGs of which the abundances increased faster in the MDCF-2 group compared with in the RUSF group were significantly enriched for those positively associated with WLZ (*q* = 3.41 × 10^−^^3^, gene set enrichment analysis (GSEA); Fig. 1c). By contrast, MAGs with a higher mean abundance as well as those that increased more rapidly in RUSF-treated children were significantly enriched for those negatively associated with WLZ (*q* = 1.57 × 10^−9^ and *q* = 3.41 × 10^−3^, respectively; GSEA) (Fig. 1c and Supplementary Table 4).

We used a ‘subsystems’ approach adapted from the SEED genome annotation platform^14,15^ to identify genes that comprise metabolic pathways represented in WLZ-associated MAGs. To do so, genes were aligned to a reference collection of 2,856 human gut bacterial genomes that had been subjected to in silico reconstructions of metabolic pathways reflecting major nutrient biosynthetic and degradative capabilities in mcSEED, a microbial community-centred implementation of SEED^16^. We used this reference collection and the procedures described in Supplementary Fig. 1 and the Methods to assign putative functions to a subset of 199,334 proteins in the 1,000 MAGs (Supplementary Table 5); these proteins, which represented 1,308 non-redundant functions, formed the basis for predicting which of 106 metabolic pathways were present or absent in each MAG. This effort generated a set of inferred metabolic phenotypes for each MAG (Supplementary Tables 6 and 7). GSEA disclosed multiple metabolic pathways that are involved in carbohydrate utilization that were significantly enriched in WLZ-associated MAGs (*q* < 0.05) and in MAGs ranked by their changes in abundance in response to MDCF-2 compared with RUSF treatment. Although other non-carbohydrate pathways were also identified using this approach (for example, those involved in aspects of amino acid and bile acid metabolism), pathways involved in carbohydrate utilization predominated (*P* = 0.006, Fisher’s test; Extended Data Fig. 2b and Supplementary Table 8).

## Glycan composition of MDCF-2 and RUSF

Before analysing the transcriptional responses of MAGs to each nutritional intervention, we characterized the carbohydrates present in MDCF-2 and RUSF, as well as their constituent ingredients (chickpea flour, soybean flour, peanut paste and mashed green banana pulp in the case of MDCF-2; rice, lentil and milk powder in the case of RUSF (Supplementary Table 9a)). Ultrahigh-performance liquid chromatography–triple quadrupole mass spectrometry (UHPLC–QqQ-MS) was used to quantify 14 monosaccharides and 49 unique glycosidic linkages. Polysaccharide content was defined using a procedure in which polysaccharides were chemically cleaved into oligosaccharides, after which the structures of these liberated oligosaccharides were then used to characterize and quantify their ‘parent’ polysaccharide^17^.

**Fig. 2:**
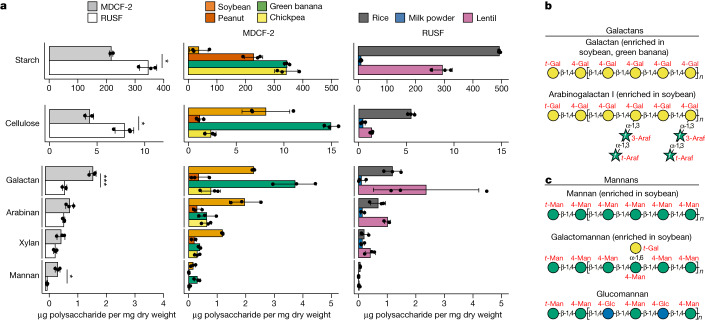
Polysaccharides in MDCF-2, RUSF and their component food ingredients. **a**, The principal polysaccharides in MDCF-2, RUSF and their component food ingredients. Data are mean ± s.d. *n* = 3 measurements of each food sample. Statistical analysis was performed using two-sided *t*-tests; *P < 0.05, ***P < 0.001. Points depict technical replicates. **b**,**c**, The structures of galactans (**b**) and mannans (**c**) in MDCF-2. f, furanose.

**Extended Data Fig. 3:**
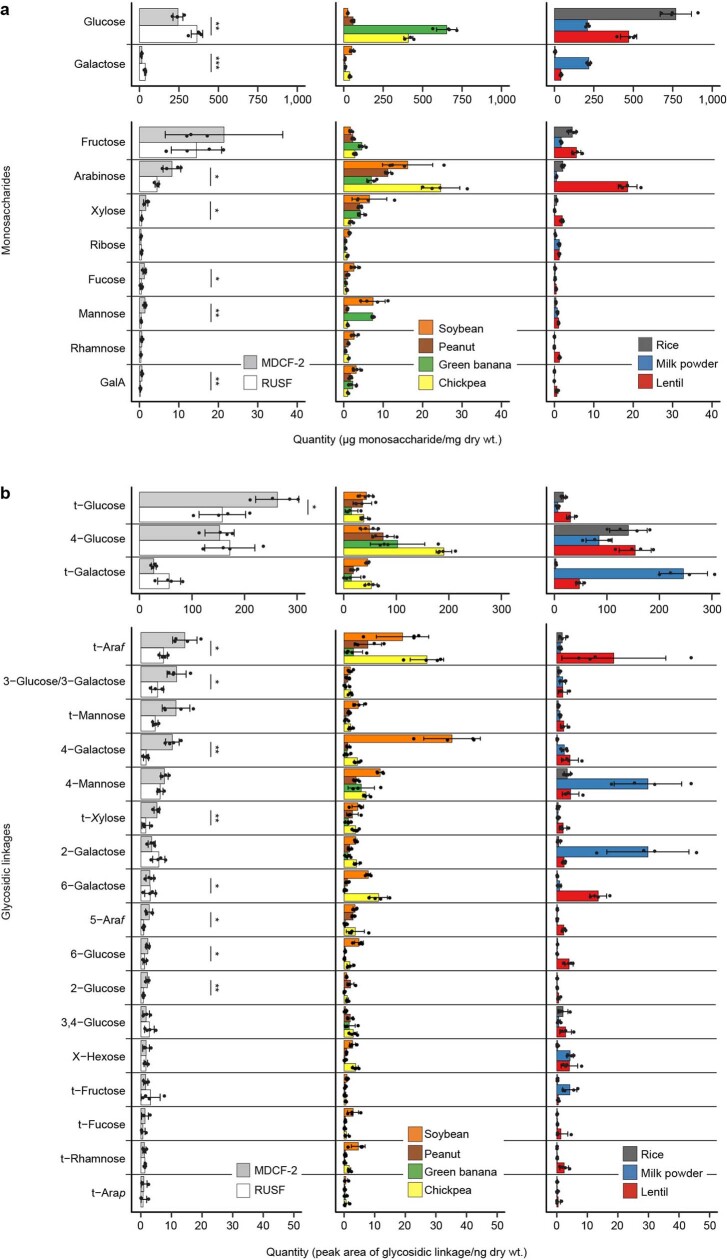
LC-MS analysis of glycans present in MDCF-2, RUSF and their component ingredients. **(a, b)** Analysis of monosaccharides (panel a) and glycosidic linkages (panel b) liberated by hydrolysis of glycans present in MDCF-2 and RUSF, and in the food ingredients used to formulate them. Mean±SD are plotted for n = 4 independent samples. *, *P* < 0.05, **, *P* < 0.01 (t-test, two-sided). Points depict individual samples.

The results revealed that l-arabinose, d-xylose, l-fucose, d-mannose and d-galacturonic acid (GalA) are significantly more abundant in MDCF-2 (*P* < 0.05; *t*-test), as are 14 linkages, eight of which contain these monosaccharides (Extended Data Fig. 3 and Supplementary Table 9b,c,e,f). Integrating the quantitative polysaccharide and glycoside linkage data enabled us to conclude that MDCF-2 contains a significantly greater abundance of galactans and mannans compared with RUSF (*P* *<* 0.05; *t*-test), whereas RUSF contains significantly more starch and cellulose (*P* < 0.05; *t*-test) (Fig. 2a and Supplementary Table 9d,g). Galactans are represented in MDCF-2 as unbranched β-1,4-linked galactan as well as arabinogalactan I (Fig. 2b). Mannans are present as unbranched β-1,4-linked mannan (β-mannan), galactomannan and glucomannan (Fig. 2c). Arabinan is abundant in both formulations, although the representation of arabinose and glycosidic linkages containing arabinose is significantly greater in MDCF-2 than in RUSF (the results of statistical tests are shown in Extended Data Fig. 3 and Supplementary Table 9e,f). Arabinan in MDCF-2 is largely derived from its soybean, banana and chickpea components, whereas, in RUSF, this polysaccharide originates from rice and lentil (Fig. 2a). Arabinans in both formulations share a predominant 1,5-linked-l-arabinofuranose (Araf) backbone. Soybean arabinans are characterized by diverse side chains composed of 1,2- and 1,3*-*linked-l-Araf connected by 1,2,3-, 1,2,5- and 1,3,5-l-Araf branch points, whereas chickpea, lentil and banana arabinans primarily contain 1,3-linked side chains from 1,3,5-l-Araf branch points^18^ (Supplementary Fig. 3).

## MDCF-2 effects on MAG gene expression

Microbial RNA-sequencing (RNA-seq) analysis was performed using RNA isolated from faecal samples collected from all of the study participants just before initiation of treatment, and after 1 month and 3 months of treatment (*n* = 350 samples). Transcripts were then quantified by mapping reads from each sample to MAGs. The resulting counts tables were filtered on the basis of the abundance and prevalence of MAGs in the full set of all faecal samples. These filtering steps were designed to exclude MAGs with minimal contributions to the metatranscriptome from subsequent differential expression analysis (exclusion criteria were benchmarked against a simulated metatranscriptomic dataset using the approach described in the Methods).

**Fig. 3:**
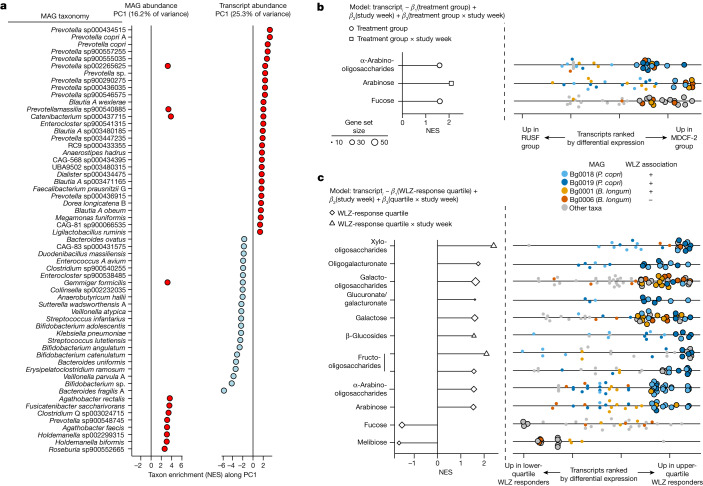
Principal taxonomic features and expressed functions of the faecal microbiomes of MDCF-2- and RUSF-treated individuals. **a**, Significant enrichment of taxa (*q* < 0.1; GSEA) along PC1 of MAG abundance or transcript abundance. NES, normalized enrichment score. **b**, Carbohydrate-utilization pathways significantly enriched (*q* < 0.1; GSEA) by treatment group (*β*_1_, circles) or the interaction of treatment group and study week (*β*_3_, squares). Right, each point represents a MAG transcript assigned to each of the indicated functional pathways (rows), ranked according to the direction and statistical significance of their differential expression in MDCF-2 versus RUSF treated participants (defined as the direction of the fold change × −log_10_[*P*]). Transcripts are coloured by their MAGs of origin. The larger, black outlined circles indicate leading-edge transcripts assigned to the pathway described on the left. **c**, Carbohydrate-utilization pathways significantly enriched (*q* < 0.1; GSEA) in upper- versus lower-quartile WLZ responders (*β*_1_, diamonds), or the interaction between WLZ-response quartile and study week (*β*_3_, triangles) (see linear mixed-effects model in the Methods section ‘Microbial RNA-seq analysis of MAG gene expression’). Right, transcripts assigned to each functional pathway. The colouring and outlining of circles have the same meaning as in **b**. The enrichment of glucuronate and galacturonate pathways was driven by the same transcripts; these pathways were therefore considered to be a single unit. Supporting information is provided in Supplementary Tables 10–14.

**Extended Data Fig. 4:**
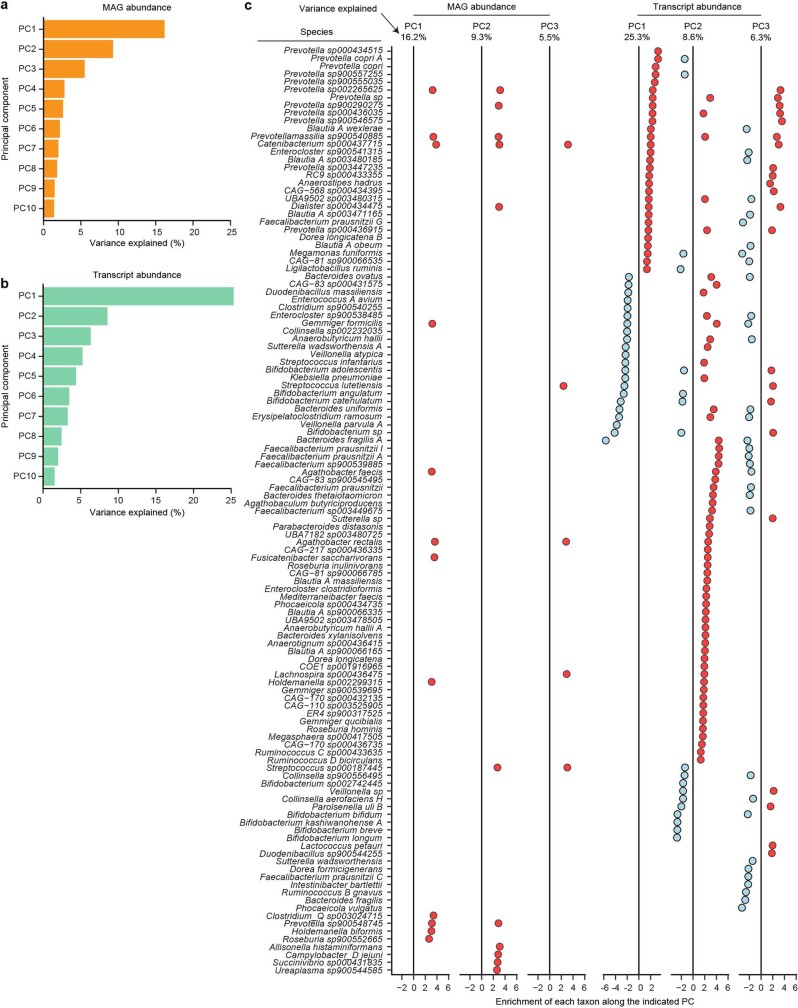
Principal components analysis of transcript and MAG abundances in faecal specimens. (**a, b**) Percent variance explained by the top 10 principal components of a PCA analysis including abundance of MAGs (panel a) at the 0, 2, 4, 8, 12, and 16 week time points in Fig. 1a, or transcripts (panel b) at 0, 4, and 12 weeks. (**c**) Significantly enriched taxa (q < 0.05, GSEA) along the first three principal components (PC1-PC3) of the faecal microbiome or meta-transcriptome PCA.

We used principal component analysis (PCA) to determine baseline differences in overall (DNA-based) MAG abundance profiles, or the abundance of MAG-derived RNAs in the expressed metatranscriptomes, between the treatment groups, and to subsequently identify microorganisms that were the principal drivers of shifts during treatment. Figure 3a shows (1) the percentage variance explained by the first principal component (PC) in analyses of 837 MAGs in faecal samples collected across all of the timepoints from all of the study participants and (2) taxa enriched (*q* < 0.05; GSEA) along the first PC of the MAG abundance and metatranscriptome datasets (Fig. 3a; details of analyses of additional PCs are shown in Extended Data Fig. 4). There were no statistically significant differences in the microbiome or metatranscriptome configuration between groups before treatment, or between the MDCF-2 and RUSF groups at each study week (*P* > 0.1; permutational analysis of variance). Analysis of MAG contributions to each PCA highlights the marked enrichment of *Prevotella* spp. transcripts and, to a lesser extent, *Bifidobacterium* spp. transcripts along the principal axis of variation (PC1) of the RNA-based PCA, and, to a much lesser degree, the enrichment of these organisms along PC1 of the DNA-based MAG abundance PCA (Supplementary Table 10).

We subsequently focused on transcripts expressed by the 222 MAGs of which the abundances were significantly associated with WLZ. Transcripts were ranked by their response to MDCF-2 versus RUSF treatment or by their response over time (negative binomial generalized linear model; Fig. 3b (equation)). GSEA was then performed to identify metabolic pathways that were enriched in these ranked transcripts. The analysis revealed an MDCF-2-associated pattern of gene expression characterized by significant enrichment (*q* < 0.1; GSEA) of three metabolic pathways related to carbohydrate utilization (α-arabinooligosaccharide (aAOS), arabinose and fucose; Fig. 3b), three pathways related to de novo amino acid synthesis (arginine, glutamine and lysine) and one pathway for de novo vitamin synthesis (folate; Supplementary Tables 11 and 12). By contrast, none of the 106 metabolic pathways exhibited statistically significant enrichment in their expression in children who received RUSF.

We next investigated which MAGs were responsible for the observed enrichment of expressed pathways. To do so, we turned to ‘leading-edge transcripts’, a term defined by GSEA as those transcripts that are responsible for enrichment of a given pathway (Methods). Among the positively WLZ-associated MAGs, two MAGs belonging to *P. copri* (MAG Bg0018 and MAG Bg0019) were the source of 11 out of the 14 leading-edge transcripts related to aAOS utilization (Supplementary Table 12)—a pathway of which expression was significantly elevated in children treated with MDCF-2 compared with RUSF (Fig. 3b). Of the 11 *P. copri* MAGs in our dataset, these two were the only MAGs assigned to this species of which the abundances were significantly positively correlated with WLZ. Both MAGs are members of a *P. copri* clade (clade A) that is broadly distributed geographically^19,20^ (Supplementary Fig. 4a; see Supplementary Fig. 4b for the predicted carbohydrate-utilization pathways represented in all 51 MAGs assigned to the genus *Prevotella* that were identified in our 1,000-MAG dataset).

Although *P. copri* MAGs were the greatest source of leading-edge transcripts related to arabinose and aAOS utilization, other MAGs in the microbiome display expression responses consistent with their participation in metabolizing MDCF-2 glycans (or their breakdown products); these include MAGs that are negatively correlated with WLZ. For example, leading-edge transcripts assigned to aAOS, arabinose and fucose utilization also arose from MAGs assigned to *Bifidobacterium longum* ssp. *longum* (Bg0006), *Bifidobacterium longum* ssp. *suis* (Bg0001), *Bifidobacterium breve* (Bg0010; Bg0014), *Bifidobacterium* sp. (Bg0070) and *Ruminococcus gnavus* (Bg0067) (Supplementary Table 12). Features of the metabolism of these glycans in *Bifidobacterium* and *Ruminococcus* MAGs are distinct from those expressed by the *P. copri* MAGs. For example, *B. longum* ssp. *longum* MAG Bg0006 encodes an extracellular exo-α-1,3-arabinofuranosidase that belongs to glycoside hydrolase (GH) family 43_22; this enzyme cleaves terminal 1,3-linked-l-Araf residues present at the ends of branched arabinans and arabinogalactans, two abundant glycans found in MDCF-2^21,22^ (Fig. 2b and Supplementary Fig. 2). By contrast, *P. copri* possesses an endo-α-1,5-l-arabinanase that cleaves interior α-1,5-l-Araf linkages, generating aAOS. Integrating these predictions suggests a complex set of interactions between primary arabinan degraders such as *P. copri* and members of *B. longum*, such as Bg0001 and Bg0006, that have the ability to metabolize products of arabinan degradation (see Supplementary Fig. 5 for reconstructions of carbohydrate-utilization pathways in *Bifidobacterium* MAGs). We cannot discern whether the arabinose available to *Bifidobacterium* is derived from free arabinose or the breakdown products of arabinan polysaccharides. It is important to consider that, in these 12- to 18-month-old children with MAM, responses to MDCF-2 are occurring in the context of the underlying co-development of their microbial community and host biology, during the period of transition from exclusive milk feeding to a fully weaned state. A MAG defined as positively associated with WLZ by linear modelling is an organism of which the fitness (abundance) increases as WLZ increases. Our studies in healthy 1- to 24-month-old children living in Mirpur have documented how *B. longum* and other members of *Bifidobacterium* decrease in absolute abundance during the period of complementary feeding^23^. For the negatively WLZ-associated *Bifidobacterium* MAGs described above, the levels of consumption of MDCF-2 metabolic products during the period of complementary feeding may not be sufficient to overcome a more dominant effect exerted on their abundance/fitness by the state of community–host co-development. Moreover, the metabolic capacities of *B. longum* including, as well as beyond, those related to the processing of MDCF-2 glycans, may influence host growth despite *B. longum* being naturally depleted over developmental time.

On the basis of these observations, we sought further evidence that the two *P. copri* MAGs are related to the magnitude of ponderal growth responses and to levels of faecal glycan structures generated from MDCF-2 metabolism.

## Glycan utilization and clinical responses

### PUL conservation in *P. copri* MAGs

As noted above, the primary outcome measure of the clinical trial was the rate of change of WLZ over the 3-month intervention. We stratified participants receiving MDCF-2 into WLZ-response quartiles^4^ and focused our analysis on (1) children in the WLZ-response upper and lower quartiles (*n* = 15 per group) and (2) transcripts expressed by the 222 MAGs of which the abundances were significantly associated with WLZ. We tested for enrichment of carbohydrate-utilization pathways in transcripts rank-ordered by the strength and direction of their relationship with WLZ-response quartile or, in a separate analysis, the interaction between the WLZ-response quartile and the study week. We next performed GSEA to identify enriched pathways (Supplementary Tables 13 and 14). Eight carbohydrate-utilization pathways were significantly enriched in transcripts that were differentially expressed in the upper- compared with lower-quartile WLZ responders. One of these pathways (fructooligosaccharide utilization) as well as three other pathways that are involved in arabinose, β-glucoside and xylooligosaccharide utilization were enriched in transcripts with a positive ‘WLZ quartile × study week’ interaction coefficient (*β*_3_), suggesting that the extent of the difference in expression of these pathways increases over the course of treatment (Fig. 3c and Supplementary Table 14a; see Supplementary Table 14b for enrichment of expressed vitamin and amino acid biosynthetic pathways related to WLZ-response quartile).

Notably, over half of the leading-edge transcripts (67 out of 99; 68%) from the eight, WLZ-response upper-quartile-enriched carbohydrate-utilization pathways were expressed by *P. copri* MAGs Bg0018 and Bg0019. Moreover, these two MAGs contributed no leading-edge transcripts to WLZ-response lower-quartile-enriched pathways.

**Fig. 4:**
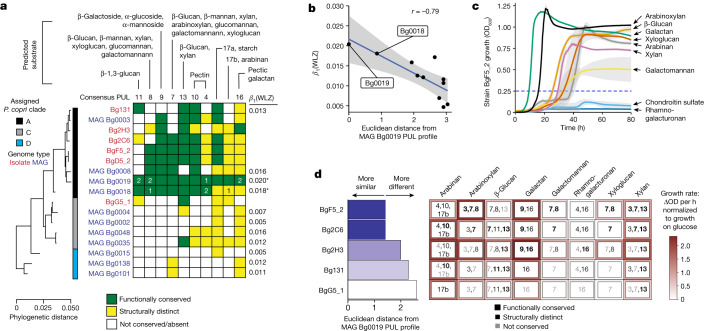
Conservation and expression of PULs in *P. copri* MAGs and isolates. **a**, PUL conservation in *P. copri* MAGs identified in study participants (blue font) and in *P. copri* isolates cultured from Bangladeshi children (red font). The marker-gene-based phylogenetic tree (left) indicates the relatedness of *P. copri* MAGs and isolates. The *β*_1_(WLZ) coefficient for each MAG is shown on the right; significant associations (*q* < 0.05) are indicated by asterisks. The matrix in the centre depicts PUL conservation among *P. copri* MAGs and cultured isolates relative to Bg0019. The number of differentially expressed PUL transcripts in MAGs Bg0018 and Bg0019 are shown within the coloured cells (identified from comparisons of MDCF-2- versus RUSF-treated participants, and/or from MDCF-2-treated participants in the WLZ-response upper versus lower quartiles; transcript annotations are shown in Supplementary Table 15). **b**, The relationship between PUL conservation in the 11 *P. copri* MAGs identified in study participants and the association of each MAG abundance with WLZ. The grey ribbon indicates the 95% confidence interval. **c**,**d**, In vitro growth assays for five *P. copri* isolates in defined medium supplemented with individual purified glycans representative of those in MDCF-2. *n* = 3 replicates per condition; two independent experiments were performed; representative results from one are shown. **c**, The results obtained with *P. copri* BgF5_2, the isolate of which the PUL profile is most similar to MAGs Bg0019/Bg0018. Data are the mean ± s.d. (grey ribbons) optical density at 600 nm (OD_600_). **d**, Summary of PUL conservation and growth rates for the five *P. copri* strains tested (Extended Data Fig. 5a). Each coloured box lists PULs in each strain (rows) that are predicted to metabolize each carbohydrate. PULs are denoted as functionally conserved (black, bold), structurally distinct but functionally similar (black, not bold) or not conserved (grey) according to the scheme shown in **a**. The colour intensity surrounding each box indicates the mean maximum growth rate for each isolate in the presence of each glycan.

*P. copri* is a member of the phylum Bacteroidota. Members of this phylum contain syntenic sets of genes known as polysaccharide-utilization loci (PULs) that mediate the detection, import and metabolism of a specific glycan or set of glycans^24^. To further define how expressed genomic features distinguish the capacity of MAGs Bg0018 and Bg0019 to respond to MDCF-2, we identified PULs in these MAGs and compared them to PULs present in the nine other *P. copri* MAGs in this study. These two WLZ-associated *P. copri* MAGs share (1) seven PULs that we designated as conserved (that is, pairwise comparisons of open reading frames (ORFs) satisfy the requirements that their protein products have greater than 90% amino acid identity and are organized in an identical way within the respective genomes); and (2) three PULs designated as present but structurally distinct (that is, a given PUL is present in the genomes being compared but component CAZymes or SusC/SusD proteins are missing or fragmented in a way that is likely to affect their function, or where extra ORFs are present; Methods).The representation of these 10 PULs varied among the other nine *P. copri* MAGs, which span three of the four principal clades of this organism (Fig. 4a and Supplementary Table 15). Notably, the degree of genomic conservation of these PULs is significantly associated with the strength of WLZ association for each of the 11 *P. copri* MAGs in our MAG dataset across both treatment groups (Pearson *r* between the Euclidean distance from Bg0019 PUL profile and *β*_1_(MAG) = −0.79 (*P* = 0.0035); Fig. 4b and Supplementary Table 3 (WLZ associations)). Five of the seven highly conserved PULs are related to utilization of mannan and galactan—glycans that are significantly more abundant in MDCF-2 than RUSF. Expression of three of these seven PULs, as well as two of the conserved but structurally distinct PULs, is also related to the enrichment of transcripts in carbohydrate-utilization pathways that distinguish upper- from lower-quartile WLZ responders (‘WLZ-response quartile’ or ‘WLZ quartile × study week’ terms in Fig. 3c). PULs that generate these leading-edge transcripts are predicted to metabolize β-glucan, glucomannan, β-mannan, xylan, pectin/pectic galactan and arabinogalactan (Fig. 4a shows which of these 10 PULs contribute differentially expressed transcripts; Supplementary Table 15).

A comparative analysis of MAGs Bg0018 and Bg0019 and 22 reference *P. copri* genomes in PULDB^25^ indicated that one of the highly conserved PULs (PUL7) contains a bimodular GH26|GH5_4 β-glycanase with 52% amino acid sequence identity to an enzyme that is known to cleave β-glucan, β-mannan, xylan, arabinoxylan, glucomannan and xyloglucan^26,27^ (Fig. 4a and Supplementary Table 15). The gene encoding this multifunctional enzyme did not satisfy our criteria for statistically significant differential expression between MDCF-2 and RUSF treatment, nor between upper- versus lower-quartile WLZ responders. However, it was consistently expressed across these conditions and comparisons (Supplementary Table 15) and its enzymatic product is expected to contribute to the utilization of a broad range of plant glycans, including those represented in MDCF-2.

Together, these results highlight both the versatility in carbohydrate metabolic capabilities of these two WLZ-associated *P. copri* MAGs, as well as the specificity of their treatment-inducible metabolic pathways for carbohydrates prominently represented in MDCF-2.

### Effect of carbohydrates on growth of *P. copri* isolates

**Extended Data Fig. 5:**
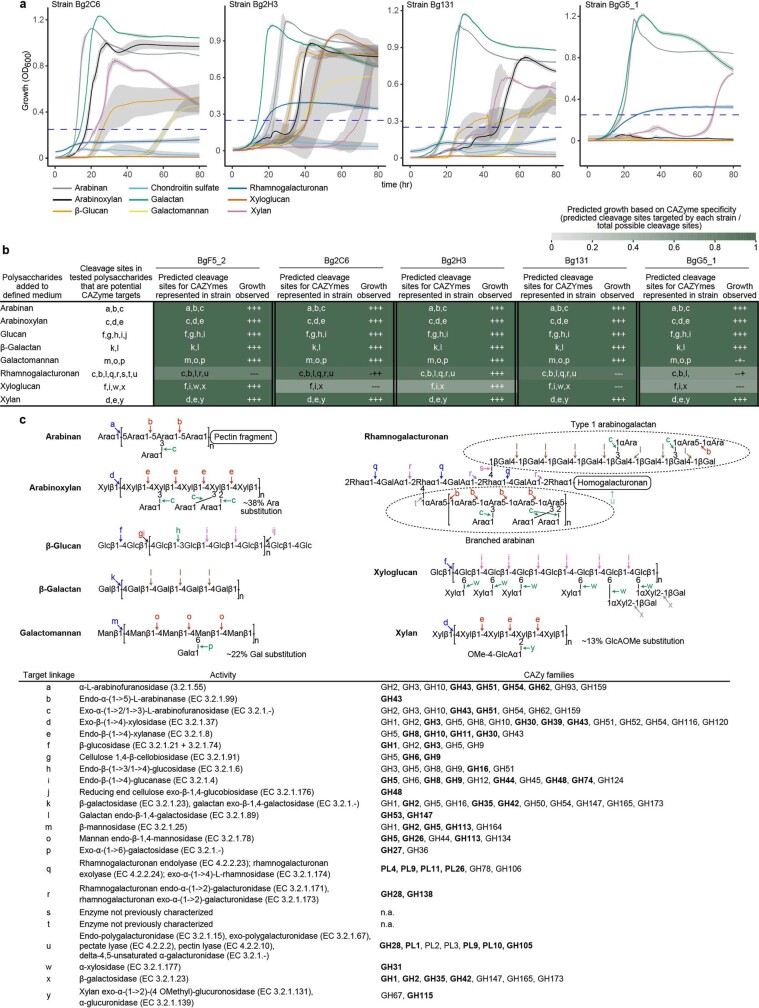
Growth of Bangladeshi *P. copri* strains in defined medium supplemented with purified polysaccharides. (**a**) Growth curves in defined medium containing individual purified polysaccharides similar to those that are abundant in or unique to MDCF-2 compared to RUSF. Curves describe mean values ± sd for OD_600_ measurements (n = 3 replicates/growth condition). (**b**) Growth of *P. copri* strains versus a PUL-based prediction of their growth phenotypes. Growth is expressed as ‘+’ or ‘-’ for each of the triplicate cultures according to whether a threshold OD_600_ > 0.25 was attained. The colour key expresses ‘prediction’ as the fraction of possible cleavage sites in each polysaccharide that are known or predicted to be targeted by the PUL-associated CAZymes of a given strain. (**c**) CAZymes present in the PULs of each strain and their predicted activities against glycosidic linkages in each purified polysaccharide. Linkages in a polysaccharide that are predicted to be targeted by PUL-associated CAZymes are labelled ‘a,b…y’ in both panels b and c. All CAZyme family assignments for a given enzymatic activity are shown; the family that displays this activity most commonly is noted in bold font.

**Extended Data Fig. 6:**
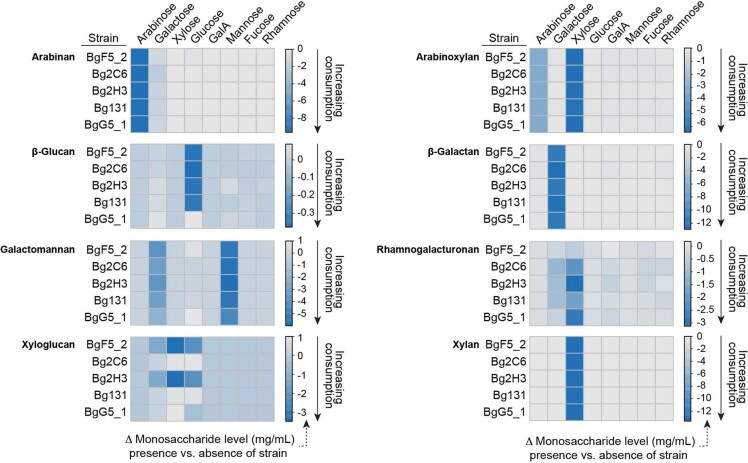
LC-MS analysis of monosaccharide consumption during growth of cultured Bangladeshi *P. copri* strains in defined medium supplemented with purified polysaccharides. Heatmaps representing bacterial consumption of monosaccharides present in the different polysaccharides. UHPLC-QqQ-MS-based monosaccharide analysis was performed using defined medium harvested from monocultures of the *P. copri* stains. Control incubations did not contain added bacteria. Cells in each matrix show the mean difference (at the end of the 189 hour-long incubation) between the concentration of each monosaccharide in three technical replicates of each strain/polysaccharide combination compared to the corresponding uninoculated control.

To contextualize our observations regarding conserved polysaccharide degradation features of *P. copri* MAGs, we engaged in an extensive effort to culture and characterize representatives of these MAGs from faecal samples obtained from the participants in this clinical trial, plus a previous, shorter duration pilot study of MDCF prototypes^3^. Based on this effort, we selected a set of six *P. copri* isolates that represented diverse repertoires of conserved PULs as well as a range of phylogenetic distances from the WLZ-associated MAGs Bg0018 and Bg0019 (Fig. 4a and Supplementary Table 1b). Strains BgD5_2 and BgF5_2 are highly related phylogenetically to each other and to MAGs Bg0018 and Bg0019. Notably, they possess 9 of the 10 conserved PULs in these MAGs (see Supplementary Tables 7c and 15b for more details of the functional conservation between the genomes of these and the other cultured *P. copri* strains and MAGs). On the basis of the substrate predictions for each conserved PUL, the measured glycan components of MDCF-2, and the variation in conservation of these PULs across our *P. copri* MAGs and isolates, we selected eight candidate glycan substrates for in vitro screening: sugar beet arabinan, wheat arabinoxylan, barley β-glucan, potato galactan, carob galactomannan, soybean rhamnogalacturonan, tamarind xyloglucan and beechwood xylan (Supplementary Table 16a). Chondroitin sulfate was included in the panel as a negative control given its resistance to degradation by *P. copri*^28^. Each cultured isolate was grown in a defined medium containing 1% (w/v) of each glycan as the sole carbon source, and growth was determined by tracking the OD over time (Fig. 4c and Extended Data Fig. 5a). Strain BgD5_2 displayed poor and inconsistent growth in this medium compared with BgF5_2, even when glucose was used as the sole carbon source; therefore, the BgD5_2 isolate was not included in these in vitro experiments. The results underscore the broad glycan-utilization abilities of the *P. copri* isolates but also highlight their distinct preferences for individual glycans. Figure 4c,d, Extended Data Figs. 5 and 6 and Supplementary Table 16b–d demonstrate that the growth phenotypes of these isolates are aligned with their PUL repertoires; show the known and predicted substrate specificities of the carbohydrate-active enzymes (CAZymes) encoded by their PULs; and present the results of MS-based quantification of their consumption of monosaccharide components of the tested glycans. Isolates of which the PUL profiles matched the two WLZ-associated MAGs most closely (BgF5_2, Bg2C6, Bg2H3) displayed the strongest preference for glycan substrates that were enriched in and/or unique to MDCF-2 relative to RUSF, including arabinans (arabinan, arabinoxylan) and galactans and mannans (galactan, galactomannan) (Fig. 2 and Supplementary Fig. 2). Notably, strain BgF5_2 displayed growth preferences for arabinoxylan and galactan; whereas all of the other strains favoured arabinan over arabinoxylan. Together, these results support predictions of the abilities of the two WLZ-associated MAGs to utilize MDCF-2 glycans; they also indicate that BgF5_2 could be considered to be a cultured representative of Bg0018 and Bg0019 given its similar glycan utilization preferences/capacities to those predicted for these two MAGs.

### Faecal glycosidic linkage levels and WLZ responses

**Fig. 5:**
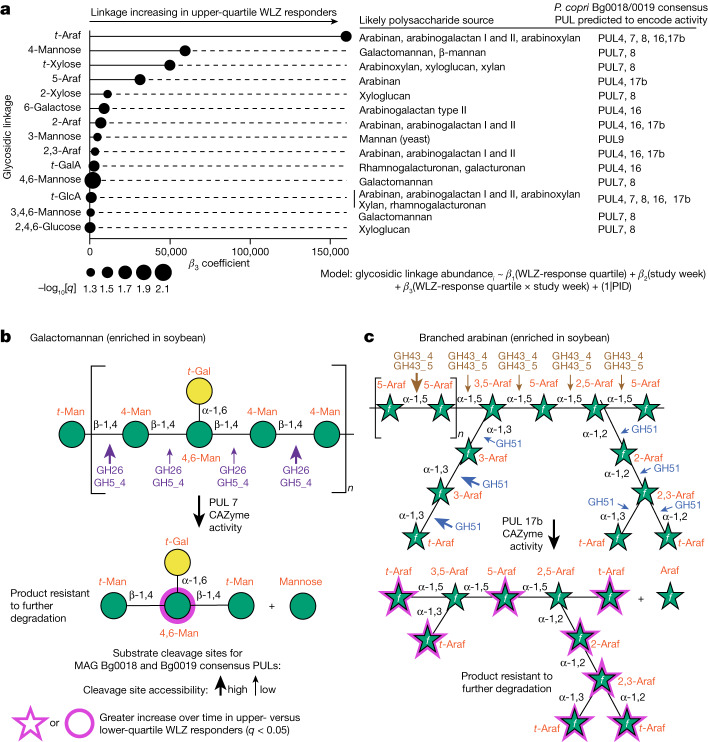
Treatment-responsive glycosidic linkages, structures of their polysaccharide sources, cleavage sites and predicted products of CAZyme activity. **a**, Significant changes in faecal glycosidic linkage levels (*q* < 0.05) over time in upper- compared with lower-quartile WLZ responders. Probable polysaccharide sources for each of the 14 glycosidic linkages are noted in the middle (Supplementary Fig. 3). PULs present in *P. copri* MAGs Bg0018 and Bg0019 with known or predicted cleavage activity for the listed polysaccharide sources are noted on the right. **b**,**c**, The structures of the MDCF-2 polysaccharides galactomannan (**b**) and branched arabinan (**c**), plus glycan fragments and their constituent glycosidic linkages predicted to be liberated by PULs conserved between *P. copri* MAGs Bg0019 and Bg0018 (the results of PUL conservation analysis are shown in Fig. 4a). The arrows indicate putative sites of cleavage by CAZymes according to their known or predicted enzyme activities. The size of each arrow (large versus small) denotes the relative likelihood (high versus low, respectively) of glycosidic linkage cleavage by the indicated CAZymes, considering steric hindrance at glycan branch points.

**Extended Data Fig. 7:**
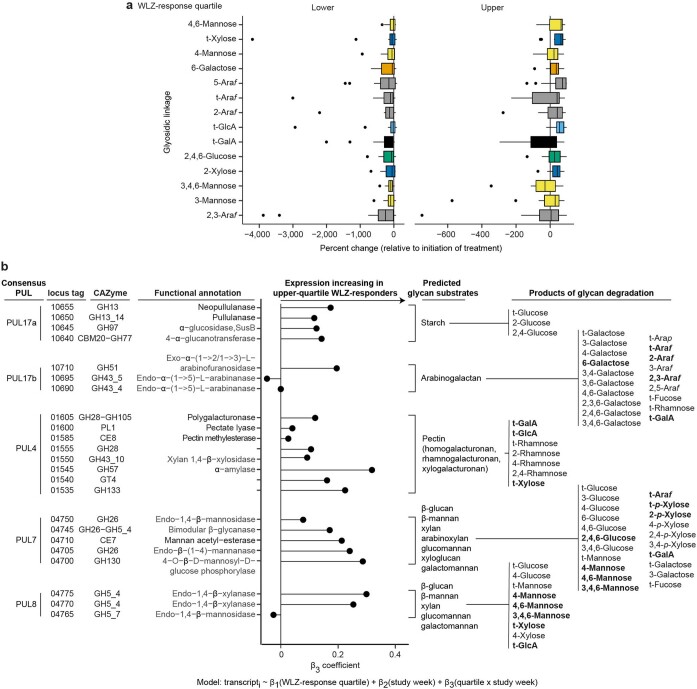
Changes in levels of faecal glycosidic linkages and expression of *P. copri* CAZyme genes after MDCF-2 treatment. (**a**) Boxplot of changes in the levels of faecal glycosidic linkages relative to initiation of treatment among upper and lower WLZ quartile responders. Levels of these 14 linkages increased to a significantly greater extent over time in the upper vs lower WLZ response quartiles (Model: linkage abundance ~ WLZ-response quartile × study week + (1|PID)). Boxplots indicate the median, first and third quartiles; whiskers extend to the largest value no further than 1.5 × the interquartile range for 90 biologically independent faecal samples obtained from n = 15 participants assigned to the upper quartile and n = 15 participants assigned to the lower quartile of WLZ-responses (n = 3 samples/participant). (**b**) The β_3_ coefficient for the interaction of WLZ-response quartile and study week is shown for CAZymes in consensus PULs in Bg0018 and Bg0019. Predicted PUL substrates and potential glycosidic linkages in each of these substrates are shown on the right. Glycosidic linkages whose abundances were significantly different in faecal samples from the upper versus lower WLZ quartile responders are highlighted in bold font (see Fig. 5a).

The same faecal samples used for the DNA- and RNA-level analyses were processed for UHPLC–QqQ-MS-based quantification of 49 glycosidic linkages. This analysis focused on samples collected at the 0- and 3-month timepoints from MDCF-2-treated individuals in the upper and lower quartile of WLZ response. These linkages were measured after their liberation by in vitro hydrolysis of faecal glycans (Supplementary Table 17). We used linear mixed-effects modelling to compare the changes in levels of faecal glycosidic linkages from the baseline/pre-intervention to the treatment endpoint (3 months) as a function of WLZ-response quartile. The results demonstrated that, with treatment, the levels of 14 linkages increased significantly more (*q* < 0.05) in members of the WLZ-response upper compared with lower quartile. None of the 49 linkages increased significantly more in children who were in the lower compared with upper quartile of WLZ response (Fig. 5a, Extended Data Fig. 7a and Supplementary Table 18a). All 14 glycosidic linkages that were elevated in upper-quartile responders are represented in MDCF-2 (for example, 4,6-mannose, which is predicted to be a product of soybean galactomannan cleavage by endo-1,4-β-mannosidases encoded by PUL7 and PUL8 present in the two WLZ-associated MAGs (Fig. 5b and Supplementary Table 9c); the likely polysaccharide sources of these 14 linkages in MDCF-2 are shown in Fig. 5a).

**Extended Data Fig. 8:**
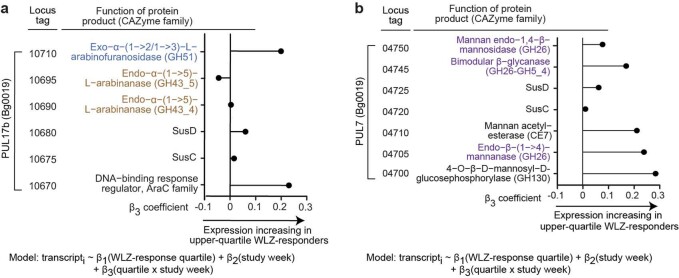
Treatment-responsive glycosidic linkages and corresponding polysaccharide sources and structures, cleavage sites, and predicted products of CAZyme activity. (**a,b**) Predicted activities and expression of *P. copri* PUL CAZymes. Panel a depicts CAZymes assigned to PUL17b, including (i) the GH51 family CAZyme (blue) expected to cleave α-1,2- and α-1,3-linked arabinofuranose (Ara*f*) side chains and (ii) GH43_4 and GH43_5 subfamily members (brown) predicted to cleave α-1,5-Ara*f*-linked backbone of branched arabinan, yielding products containing *t*-Ara*f*, 2-Ara*f*, 2,3 Ara*f* and 5-Ara*f* linkages. Panel b depicts CAZymes assigned to PUL7, including GH26 and GH5_4 CAZymes (magenta) predicted to cleave β-1,4 linked mannose residues of galactomannan, yielding products containing 4,6-mannose which is the most significantly differentially abundant linkage in the upper quartile WLZ responders (see Fig. 5a). The CAZyme colouring scheme matches that used in Fig. 5b, c.

**Extended Data Fig. 9:**
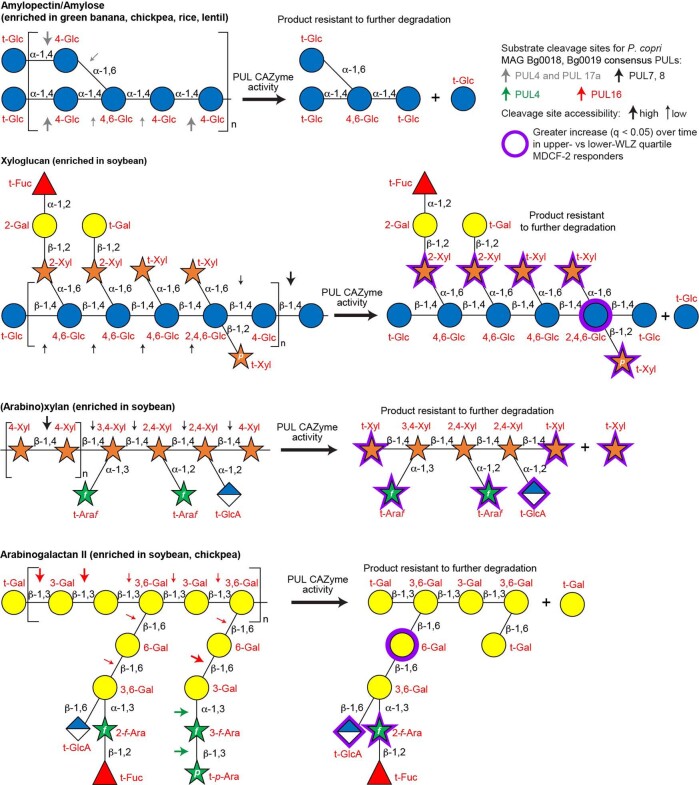
Polysaccharide structures, cleavage sites, and predicted products of CAZyme activity. Glycosidic linkages highlighted with arrows are those predicted as sites of cleavage by CAZymes expressed by the set of PULs, described in Fig. 4a, that are present in *P. copri* MAG Bg0019 and/or Bg0018. Consensus PUL numbers are listed except in the case of Bg0019 PUL3, which is not represented in Bg0018 (see Supplementary Table 15). The size of the arrows (large versus small) denotes the relative likelihood (high versus low, respectively) of cleavage of glycosidic linkages by *P. copri* CAZymes when considering steric hindrance at branch points.

The levels of glycosidic linkages in the faeces reflect a complex dynamic that includes, but is not necessarily limited to, the substrate specificities of the CAZymes encoded and expressed by PULs in primary consumers of available polysaccharides, the levels of host consumption of MDCF-2 and components of their ‘background’ diets, and the degree to which the initial products of polysaccharide degradation can be further processed by community members. These points are illustrated by the following observations. First, the presence of the 14 glycosidic linkages in the faeces can be explained in part by the specificity of CAZymes encoded and expressed by PULs conserved between *P. copri* MAGs Bg0018 and Bg0019. Figure 5b,c and Extended Data Figs. 7b, 8 and 9 describe which of their PULs are predicted to generate glycan fragments containing these linkages—predictions that are supported by the in vitro data generated from the cultured representative of the two MAGs. For example, *t*-Araf, 5-Araf, 2-Araf and 2,3-Araf are components of polysaccharides (arabinan, arabinoxylan and arabinogalactan type I/II) present in soybean, chickpea, peanut and banana. CAZymes encoded by *P. copri* Bg0019 PULs 4, 7, 8, 16 and 17b have substrate specificities that enable them to cleave accessible linkages in these polysaccharides (Fig. 5b,c and Extended Data Figs. 7b and 9). Some of the products of these cleavage events are probably resistant to further degradation. The exo-α-1,2/1,3-l-arabinofuranosidase and endo-α-1,5-l-arabinanase activities encoded by PUL17b (Fig. 5c) are predicted to remove successive residues from the 1,2- and 1,3-linked-l-Araf chains of branched arabinan and hydrolyse the 1,5-linked-l-Araf backbone from this polysaccharide, yielding an enzyme-resistant product containing *t*-Araf, 5-Araf, 2-Araf and 2,3-Araf linkages. Second, CAZyme transcripts assigned to PULs 4, 7, 8, 16 and 17b were detectable in the faecal metatranscriptomes of all but 1 of the 30 participants assigned to the WLZ-responder upper or lower quartiles. The levels of expression of the majority of these CAZymes genes were modestly elevated in upper-quartile compared with the lower-quartile WLZ responders over the course of treatment, although the difference did not reach our threshold cut-off for statistical significance (*q* < 0.05). These transcripts include the GH51 encoded by PUL17b plus the GH26, GH26|GH5_4, GH130 and carbohydrate esterase family 7 (CE7) transcripts from PUL7 (Extended Data Fig. 7b). Third, while intake of MDCF-2 was not significantly different between the upper- and lower-quartile WLZ participants (*P* > 0.05; linear mixed-effects model), data from a food frequency questionnaire administered at the time of each faecal sampling disclosed a positive correlation between consumption of legumes and nuts and the levels of *t*-Araf, 5-Araf, 2,3-Araf, *t*-GalA and 2,4,6-glucose (Supplementary Table 18b). Consumption of these foods was also the most discriminatory response between upper-quartile compared with lower-quartile WLZ responders (Supplementary Table 18c). These observations suggest that children consuming more of the classes of complementary food ingredients present in MDCF-2 may also exhibit enhanced growth responses.

The confounding effects of background diet and the role of *P. copri* in processing MDCF-2 glycans can be directly tested in gnotobiotic mice colonized with a defined community of cultured representatives of WLZ-associated MAGs. One such gnotobiotic model is described in our ‘reverse translation’ study in which mice were colonized with a defined consortium of age- and WLZ-associated bacterial strains from Bangladesh, with or without *P. copri* isolates that captured key features of the carbohydrate metabolic apparatus present in Bg0018 and Bg0019 (such as BgF5_2/BgD5_2) and fed either MDCF-2 or a diet representative of that consumed by children living in Mirpur. Analyses revealed that these *P. copri* strains were the principal mediators of MDCF-2 glycan degradation in vivo and that the combination of the presence of *P. copri* and MDCF-2 diet was associated with promoting ponderal growth and had marked effects on multiple aspects of metabolism in intestinal epithelial cell lineages^29^.

## Discussion

Here we illustrate an approach for characterizing the gut microbiome targets and structure–function relationships of a therapeutic food—in this case, MDCF-2. MDCF-2 produced significantly greater weight gain during a 3-month, randomized controlled study of 12- to 18-month-old Bangladeshi children with MAM compared with a conventional, more calorically dense RUSF. We focused on MAGs, specifically (1) treatment-induced changes in the expression of carbohydrate metabolic pathways in MAGs whose abundances were significantly associated with weight gain (WLZ); and (2) MS analysis of the metabolism of glycans present in the two food formulations. Quantifying monosaccharides, glycosidic linkages and polysaccharides present in MDCF-2, RUSF and their component ingredients revealed that MDCF-2 contains more galactans and mannans (for example, galactan, arabinogalactan I, galactomannan, β-mannan and glucomannan). Two types of comparisons were performed of the transcriptional responses of MAGs that were significantly associated with WLZ: one involved study participants who had consumed MDCF-2 versus RUSF, and the other focused on MDCF-2-treated children in the upper versus lower quartiles of WLZ response. The results revealed that two *P. copri* MAGs, both positively associated with WLZ, were the principal contributors to MDCF-2-induced expression of metabolic pathways involved in the utilization of its component glycans.

Using UHPLC–QqQ-MS, we were able to identify statistically significant changes in glycan composition in the faeces of children consuming a therapeutic food, even in the face of complex and varied background diets. Notably, although the intake of MDCF-2 did not differ between children in the upper and lower quartiles of clinical (WLZ) response, children in the upper quartile trended toward diets containing more legumes and nuts compared with those in the lower quartile. The legumes and nuts food group includes major components of MDCF-2. We postulate that MDCF-2 ‘kick starts’ a microbiome response that includes changes in the fitness and expressed metabolic functions of key growth-associated bacterial strains, such as *P. copri*. Background diet can further modify this response, as evidenced by the higher levels of microbial metabolic products of legume/nut-associated glycans in the faeces of children displaying upper-quartile WLZ responses. This observation also suggests that further optimization of the dose of MDCF-2 may be possible; in our study, MDCF-2 was administered as a dietary supplement designed to provide around 20% of the children’s daily energy requirements. More detailed, quantitative assessments of food consumption during future clinical studies of MDCF-2 could not only facilitate the design of improved formulations/doses but also inform future recommendations regarding complementary feeding practices—recommendations that recognize the important role of the gut microbiome in the healthy growth of children.

One definition of ‘microbiome repair’ in children with malnutrition is a rebalancing of the representation and expressed functions of beneficial organisms so that it assumes a configuration that is more conducive to healthy microbiome–host co-development. Linking dietary glycans and microbial metabolism in this manner provides a starting point for culture-based initiatives designed to retrieve isolates of these ‘effector’ taxa for use as potential probiotic agents, or if combined with key nutrients that they covet, synbiotic formulations for repairing perturbed microbiomes that are insufficiently responsive to food-based interventions alone.

The link between MDCF-2 treatment, *P. copri* glycan metabolism and ponderal growth is not formally established by the data described in this Article. Much remains to be discovered about how MDCF-2 treatment is related to weight gain and healthy growth. For example, further work is needed to clarify whether the mediators of *P. copri* effects on the host arise from direct products of its metabolism of MDCF-2 glycans, or whether products of other metabolic pathways in *P. copri*, of which the activities are regulated by biotransformation of MDCF-2 glycans, are involved. Moreover, the contributions of metabolites from other community members to these effects are unclear. Furthermore, the observed relationships between strains of *P. copri* and MDCF-2 glycans do not exclude the contribution of other macro- or micronutrients to the superiority of MDCF-2 over RUSF on weight gain in our study. Direct tests of the role of organisms such as *P. copri* in mediating microbial community and host responses to components of microbiome-targeted therapeutic foods can come from additional clinical studies of probiotic or synbiotic formulations consisting of strains that are closely related to WLZ-associated MAGs, such as *P. copri* BgF5_2, administered in conjunction with MDCF-2 or with its glycan components. Another approach that we are using involves reverse-translation experiments that use gnotobiotic mice (1) colonized with defined collections of various combinations of cultured, WLZ-associated gut bacterial taxa, with or without *P. copri* strains, and (2) fed diets with or without MDCF-2, or various combinations of its component glycans^29^.

## Methods

### Collection and handling of biospecimens obtained from participants in the randomized controlled clinical study of the efficacy of MDCF-2

The human study entitled ‘Community-based clinical trial with microbiota-directed complementary foods (MDCFs) made of locally available food ingredients for the management of children with primary moderate acute malnutrition (MAM)’ was approved by the Ethical Review Committee at the icddr,b (protocol PR-18073; ClinicalTrials.gov: NCT04015999)^4^. Informed consent was obtained for all of the participants. The objective of the study was to determine whether twice daily, controlled administration of a locally produced, microbiota-directed complementary food (MDCF-2^3,4^) for 3 months to children with MAM provided superior improvements in weight gain, microbiota repair and improvements in the levels of key plasma biomarkers/mediators of healthy growth, compared with a commonly used rice- and lentil-based RUSF formulation.

A total of 124 male and female children with MAM (WLZ, −2 to −3) between 12- and 18-months old who satisfied the inclusion criteria were enrolled, with 62 children randomly assigned to each treatment group using the permuted block randomization method. Children in each treatment group were fed their assigned dietary supplement (MDCF-2 or RUSF) twice daily at a study centre for the first month, once daily at a study centre and once daily at home for the second month, and twice daily at home for the third month. Mothers were otherwise encouraged to practice their customary breast-feeding and complementary-feeding practices. At the end of the intervention period, children returned to their normal feeding routine with continued intensive monitoring for one additional month. Fifty-nine participants in each treatment group completed the 3-month intervention and 1-month post-treatment follow-up^4^.

To minimize the risk of degradation of faecal DNA/RNA, faecal samples were collected within 20 min of their production and immediately transferred to liquid-nitrogen-charged vapour shippers for transport to a −80 °C freezer at the study centre. The samples were shipped to Washington University on dry ice where they were stored at −80 °C in a dedicated repository with approval from the Washington University Human Research Protection Office.

### MAGs

#### Short-read shotgun sequencing

DNA was isolated from 942 faecal samples as previously described^4^ and shotgun sequencing libraries were prepared using a reduced-volume Nextera XT (Illumina) protocol^30^. Libraries were quantified, balanced, pooled and sequenced (Illumina NovaSeq 6000, S4 flow cell; 2.3 ± 1.4 × 10^7^ 150-nucleotide paired-end reads/sample (mean ± s.d.)). Reads were demultiplexed (bcl2fastq, Illumina), trimmed to remove low-quality bases and processed to remove read-through adapter sequences (Trim Galore^31^, v.0.6.4). Read pairs for which the length of either read was <50 nucleotides after quality and adapter trimming were discarded. The remaining reads were mapped to the human genome (UCSC hg19) using bowtie2^32^ (v.2.3.4.1) and were subsequently filtered to remove *Homo sapiens* sequences.

Preprocessed, short-read shotgun data were aggregated from each participant’s faecal sample set (*n* = 7–8 samples per participant; 118 participants) before MAG assembly. This strategy was adopted to enable the contig abundance calculations required by the MAG assembly algorithms used below, while at the same time mitigating the risk of chimeric assemblies inherent to co-assembly across individuals. Assemblies were generated for all 118 datasets using MegaHit^33^ (v.1.1.4), and the resulting contigs were quantified in each assembly by mapping preprocessed reads to the assembled contigs using kallisto^34^. Contigs were assembled into MAGs using MaxBin2^35^ (v.2.2.5) and MetaBAT2^36^ (v.2.12.1). The parallel results of both binning strategies were merged and dereplicated using DAS Tool^37^ (v.1.1.2) on a per-participant basis.

#### Long-read shotgun sequencing

We applied long-read sequencing to faecal samples obtained at the 0- and 3-month timepoints from each of the 15 upper-quartile WLZ responders in the MDCF-2 treatment group. Aliquots containing 400–1,000 ng of DNA from each biospecimen were transferred to a 96-well, 0.8 ml, deep-well plate (Nunc, Thermo Fisher Scientific) and prepared for long-read sequencing using the SMRTbell Express Template Prep Kit 2.0 (PacBio). All subsequent DNA handling and transfer steps were performed with wide-bore, genomic DNA pipette tips (ART, Thermo Fisher Scientific). Barcoded adapters were ligated to A-tailed DNA fragments by overnight incubation at 20 °C. Adapter-ligated fragments were then treated using the SMRTbell Enzyme Cleanup Kit to remove damaged or partial SMRTbell templates. A high molecular mass DNA fraction was purified using AMPure beads (ratio of 0.45× well-mixed AMPure bead volume to sample) and eluted in 12 µl of PacBio elution buffer. DNA libraries were sequenced on the Sequel System (Pacific Biosciences) using the Sequel Binding Kit 3.0 and Sequencing Primer v4 with 24 h of data collection. A total of 3.0 × 10^9^ ± 9.8 × 10^8^ bp per sample were collected, with an average subread length of 5,654 ± 871 bp (mean ± s.d.).

Hybrid assembly of short- and long-read data was performed using OPERA-MS^38^ (v.0.9.0). OPERA-MS uses assembly graph and coverage-based methods to cluster contigs into MAGs based on optimizing per-cluster Bayesian information criterion. Before hybrid assembly, continuous long reads (CLRs) were combined across the two available timepoints for each participant and reads that mapped to the human genome were removed. Illumina short reads and PacBio long reads (CLRs) were provided to OPERA-MS and assembled using the built-in OPERA-MS genome database and the default settings (the latter includes polishing of output MAGs with Pilon^39^).

#### MAG dereplication, curation and abundance calculations

**Extended Data Fig. 10:**
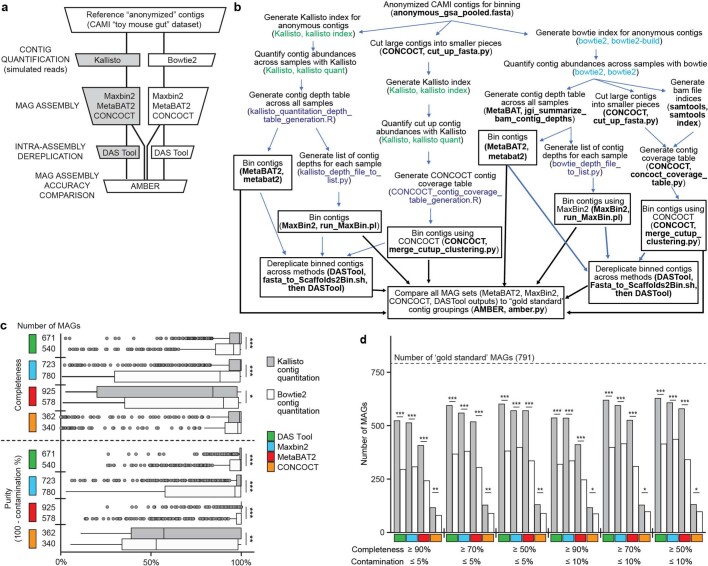
Validation of the MAG assembly pipeline. **(a)** Bioinformatic workflow for comparing the fidelity of MAG assembly from alignment-based (bowtie2) versus pseudoalignment-based (kallisto) contig quantitation. **(b)** A detailed description of the workflow is described in panel a. Each box includes a summary of the computational task plus the name of the program and, where relevant, the command used to complete the task (in parentheses). Colour of the text in parentheses: brown, default code from kallisto; purple, default code from bowtie2; blue, custom script written to achieve tasks described; black, default code used for programs to assemble MAGs and dereplicate contigs. Boxes with a black outline and the thick black arrows emanating from them indicate that binned contigs were used as input to AMBER for MAG assembly comparisons across methods. **(c)** Boxplot describing summary statistics (completeness and purity) for MAG assembly approaches. Boxplots indicate the median, first and third quartiles; whiskers extend to the largest value no further than 1.5 × the interquartile range. The number of individual MAGs generated using each MAG assembly tool-quantitation method was 671 for DAS tool-kallisto, 723 for MaxBin2-kallisto, 925 for MetaBAT2-kallisto, 362 for CONCOCT-kallisto, 540 for DAS tool-bowtie2, 780 for MaxBin2-bowtie2, 578 for MetaBAT2-bowtie2 and 340 for CONCOCT-bowtie2. **(d)** Number of MAGs, obtained from each assembly and quantitation strategy, that are distributed across two quality metrics (completeness, contamination). The number of ‘gold standard’ MAGs (theoretical maximum) in the simulated metagenomic dataset is indicated by a horizontal dashed line. *, *P* < 0.05; **, *P* < 0.01; ***, *P* < 0.001 (two-tailed, unpaired Wilcoxon test for panel c; two-tailed Fisher’s exact test for panel d).

After assembling MAGs using both the short-read-only and short-read plus long-read strategies, all MAGs from all assembly strategies were assessed for completeness and contamination (‘lineage_wf’ command in CheckM^40^, v.1.1.3) and refined (‘tetra’, ‘outliers’ and ‘modify’ commands in CheckM) to remove contaminating contigs. Additional refinement based on the distribution of phylogenetic markers present in each MAG was performed (‘phylo-markers’, ‘clade-markers’ and ‘clean-bin’ commands in MAGpurify^41^ (v.2.1.2)). A final MAG quality assessment was performed using CheckM, followed by a stringent (≥90% complete, ≤5% contaminated, average nucleotide identity (ANI) ≥ 99%) bulk dereplication across all MAGs from all of the participants (options ‘-l 50000’, ‘--completeness 90’, ‘--contamination 5’, ‘-pa 0.9’, ‘-sa 0.99’ in dRep^42^ (v.2.6.2)). The final dataset contained 681 ± 99 (mean ± s.d.) MAGs per participant. MAG assembly summary statistics were collected from CheckM^40^ and quast^43^ analyses (v.4.5) and aggregated (Supplementary Table 1 and Extended Data Fig. 10). Initial MAG annotations were performed using prokka^44^ (v.1.14.6). Further details about benchmarking the methods we used for assembly are presented in the Supplementary Discussion. To quantify the abundance of each MAG in each sample, MAGs were processed to create a single kallisto quantification index^45^. Reads from each faecal DNA sample were then mapped to this index.

#### MAG taxonomy

Taxonomic assignments were initially made using the Genome Taxonomy Database Toolkit^13^ (GTDB-Tk) and the corresponding database (release 95). We complemented these MAG assignments using Kraken2^46^ (v.2.0.8) and Bracken^47^ (v.2.5) and a Kraken2-compatible version of the GTDB reference.

*P. copri* has been partitioned into four distinct clades (A–D) on the basis of marker gene phylogeny^19^. To classify *Prevotella* MAGs in this study, we constructed an unrooted, marker-gene-based phylogeny using Phylophlan^48^ (v.3.0.60). This tree encompassed 17 reference isolate genomes and 1,006 MAGs from a previous study^19^ plus any MAGs from our set classified by GTDB-Tk as belonging to the genera *Prevotella* (*n* = 51) or *Prevotellamassilia* (*n* = 13). Putative *Prevotella* MAGs from the present study that clustered within the four previously identified *P. copri* clades were assigned to the corresponding clade based on visualization using Graphlan^49^ (v.1.1.4).

Certain *Bifidobacterium* species consist of multiple closely related subspecies (such as *B. longum*). We therefore calculated a pan-genome for 34 *Bifidobacterium* MAGs in our dataset (Supplementary Table 1), plus 14 reference isolate genomes (Supplementary Fig. 5), using Roary^50^ (v.3.12.0) and a 60% minimum sequence identity threshold for BLASTp^51^. The reference isolate genomes included ten *Bifidobacterium* species and three subspecies of *B. longum* (ssp. *longum*, *infantis* and *suis*). Concatenated nucleotide sequences of 142 identified core genes were aligned using MAFFT^52^ (v.7.313). The resulting alignment was trimmed (microseq^53^ R package (v.2.1.4)) and then used to construct a maximum-likelihood phylogenetic tree (IQ-TREE^54^ (v.1.6.12)). The *B. gallicum* DSM 20093 genome was selected as an outgroup. Putative *Bifidobacterium* MAGs from this study that clustered together with reference genome clades were assigned to the corresponding clade. Using this method, we were able to confirm or update our initial GTDB-Tk-based classifications of all *Bifidobacterium* MAGs and resolve nearly all closely related subspecies (Supplementary Table 1 and Supplementary Fig. 5).

### Defining the relationship between MAG abundances and WLZ

The procedures used for shotgun sequencing of faecal DNA and preprocessing of the resulting reads, plus MAG assembly and quantification are described in the Methods.

Linear mixed-effects models were used to relate the abundances of MAGs identified in each trial participant to WLZ using the formula:

$${\rm{WLZ}} \sim {\beta }_{1}({\rm{MAG}})+{\beta }_{2}({\rm{study}}\,{\rm{week}})+(1| {\rm{PID}})$$

Our data normalization strategies before linear modelling did not include a consideration of MAG assembly length. We therefore analysed the TPM (reads per kilobase per million) output of kallisto (v.0.43.0) by applying a filter requiring each MAG’s abundance to be >5 TPM in >40% of the 707 faecal samples collected at timepoints at which anthropometry was also measured. This filtering approach yielded 837 MAGs. We then returned to the unfiltered count output from kallisto, performed a variance-stabilizing transformation (VST, DESeq2^55^ (v.1.34.0)) to control for heteroskedasticity, and filtered the dataset to the same 837 MAGs. We subsequently fit linear mixed-effects models to the transformed abundances of each MAG across all 707 faecal samples (lme4^56^, v.1.1-27.1; lmerTest^57^, v.3.1-3). We used analysis of variance to determine the statistical significance of the fixed effects in our model—specifically, the relationship between MAG abundance and WLZ. WLZ-associated MAGs were defined as those with false-discovery-rate-adjusted *P* (*q*) < 0.05.

### Determining the effects of MDCF-2 supplementation on the abundances of WLZ-associated MAGs

We used dream^58^ (variancePartition R package, v.1.24.0) an empirical Bayesian linear mixed-effects modelling framework, to model MAG abundance as a function of treatment group, study week and their interaction, controlling for the repeated measurements taken from each study participant with a random effect term for participant. The equation used to quantify the effects of treatment on MAG abundance took the form:

$$\begin{array}{l}{{\rm{M}}{\rm{A}}{\rm{G}}}_{i} \sim {\beta }_{1}({\rm{t}}{\rm{r}}{\rm{e}}{\rm{a}}{\rm{t}}{\rm{m}}{\rm{e}}{\rm{n}}{\rm{t}}\,{\rm{g}}{\rm{r}}{\rm{o}}{\rm{u}}{\rm{p}})+{\beta }_{2}({\rm{s}}{\rm{t}}{\rm{u}}{\rm{d}}{\rm{y}}\,{\rm{w}}{\rm{e}}{\rm{e}}{\rm{k}})\\ \,\,\,+{\beta }_{3}({\rm{t}}{\rm{r}}{\rm{e}}{\rm{a}}{\rm{t}}{\rm{m}}{\rm{e}}{\rm{n}}{\rm{t}}\,{\rm{g}}{\rm{r}}{\rm{o}}{\rm{u}}{\rm{p}}\,\times \,{\rm{s}}{\rm{t}}{\rm{u}}{\rm{d}}{\rm{y}}\,{\rm{w}}{\rm{e}}{\rm{e}}{\rm{k}})+(1|{\rm{P}}{\rm{I}}{\rm{D}})\end{array}$$

The ‘treatment group’ coefficient *β*_1_ indicates whether MDCF-2 produced changes in the mean abundance of a given MAG relative to RUSF over the 3-month intervention, whereas the ‘treatment group × study week’ interaction coefficient *β*_3_ indicates whether MDCF-2 affected the rate of change of a given MAG more so than RUSF (that is, whether a MAG increases or decreases more rapidly in the microbiomes of participants in the MDCF-2- versus the RUSF-treatment group). Each coefficient for each MAG abundance analysis is described by an associated *t*-statistic—a standardized measure, based on standard error, of a given coefficient’s deviation from zero that can be used to calculate a *P* value and infer the significance of the effect of a given coefficient on the dependent variable. The *t*-statistics produced using this method can also be used as a ranking factor for input into GSEA. For this analysis, gene sets were defined as groups of MAGs that were either significantly positively (*n* = 75) or significantly negatively (*n* = 147) associated with WLZ. This analysis was conducted for both the ‘treatment group’ (*β*_1_) coefficient and the ‘treatment group × study week’ interaction (*β*_3_) coefficient. Statistical significance is reported as *q* values after adjustment for false-discovery rate (using the Benjamini–Hochberg method).

### Microbial RNA-seq analysis of MAG gene expression

For RNA extraction, approximately 50 mg of a faecal sample, collected from each participant at the baseline, 1-month and 3-month timepoints, was pulverized under liquid nitrogen with a mortar and pestle and aliquoted into 2 ml cryotubes. A 3.97 mm steel ball and 250 µl of 0.1 mm zirconia/silica beads were subsequently added to each sample tube, together with 500 µl of a mixture of phenol:chloroform:isoamyl alcohol (25:24:1, pH 7.8–8.2), 210 µl of 20% SDS and 500 µl of 2× Qiagen buffer A (200 mM NaCl, 200 mM Trizma base, 20 mM EDTA). After a 1 min treatment in a bead beater (Biospec Minibeadbeater-96), the samples were centrifuged at 3,220*g* for 4 min at 4 °C. Then, 100 μl of the resulting aqueous phase was transferred by a liquid-handling robot (Tecan) to a deep 96-well plate along with 70 µl of isopropanol and 10 µl of 3 M NaOAc, pH 5.5. The solution was mixed by pipetting ten times. The crude DNA/RNA mixture was incubated at −20 °C for 1 h and then centrifugated at 3,220*g* at 4 °C for 15 min before removing the supernatant to yield nucleic-acid-rich pellets. A Biomek FX robot was used to add 300 µl Qiagen buffer RLT to the pellets and to resuspend the RNA/DNA by pipetting up and down 50 times. A 400 µl aliquot was transferred from each well to an Qiagen AllPrep 96 DNA plate, which was centrifuged at 3,220*g* for 1 min at room temperature. The RNA flow-through was purified as described in the AllPrep 96 protocol. cDNA libraries were prepared from extracted RNA using an Illumina Total RNA Prep with Ribo-Zero Plus and dual unique indexes. Libraries were balanced, pooled and sequenced in two runs of an Illumina NovaSeq using S4 flow cells.

As an initial pre-processing step, raw reads were aggregated by sample across the two NovaSeq runs, resulting in a total of 5.0 × 10^7^ ± 4.7 × 10^6^ paired-end 150-nucleotide reads per sample (mean ± s.d.). Adapter sequences and low-quality bases were removed from raw reads (Trim Galore^31^, v.0.6.4), and pairs of trimmed reads were filtered out if either one of the paired reads was less than 100 nucleotides long. Pre- and post-trimmed sequence quality and adapter contamination were assessed using FastQC^59^ (v.0.11.7). Filtered reads were pseudoaligned to the set of 1,000 annotated, dereplicated high-quality MAGs to quantify transcripts with kallisto^34^. Reads that pseudoaligned to rRNA genes were excluded, leaving an average of 7.1 × 10^6^ ± 3.9 × 10^6^ bacterial mRNA reads (mean ± s.d.) per sample. Count tables were further filtered to retain only transcripts that pseudoaligned to the 837 MAGs that passed the abundance and prevalence thresholds described above. To minimize inconsistently quantified counts related to low-abundance MAGs, we assigned a transcript count of zero, on a per-sample basis, to any MAG with a DNA abundance < 0.5 TPM in that sample.

Differential expression analysis (edgeR^60^, v.3.32.1) was conducted using the following steps: (1) transcript filtering for presence/absence and prevalence; (2) library-size normalization using trimmed mean of *M* values (TMM); (3) estimating per-gene count dispersions; and (4) testing for differentially expressed genes. Transcripts were first filtered using the edgeR default parameters, followed by a parameter sweep of transcript abundance and prevalence threshold combinations. On the basis of this analysis, transcripts with ≥5 counts per million mapped reads in ≥35% of samples were retained for differential expression analysis. The transcripts that passed this filtering were normalized using a TMM-based scaling factor. We next estimated negative binomial dispersions and fit trended per-gene dispersions (using the power method) to negative binomial generalized linear models. These models were used to characterize (1) the effect of treatment group and study week among all participants and (2) the effect of WLZ quartile and study week among MDCF-2 participants in the upper and lower quartiles of WLZ response using the following model formula:

$$\begin{array}{l}{{\rm{transcript}}}_{i} \sim {\beta }_{1}({\rm{treatment}}\,{\rm{group}})+{\beta }_{2}({\rm{study}}\,{\rm{week}})\\ \,\,\,\,\,\,+\,{\beta }_{3}({\rm{treatment}}\,{\rm{group}}\times {\rm{study}}\,{\rm{week}})\\ {{\rm{transcript}}}_{i} \sim {\beta }_{1}({\rm{WLZ-response\; quartile}})+{\beta }_{2}({\rm{study}}\,{\rm{week}})\\ \,\,\,\,\,\,+\,{\beta }_{3}({\rm{WLZ-response\; quartile}}\times {\rm{study}}\,{\rm{week}})\end{array}$$

From these models, we identified genes that exhibited significant differential expression using the quasi-likelihood *F*-test (edgeR, function glmQLFTest), which accounts for the uncertainty in estimating the dispersion for each gene.

For subsequent functional metabolic pathway enrichment analyses, we (1) ordered transcripts assigned to WLZ-associated MAGs on the basis of a ranking metric calculated as the direction of the fold-change × −log_10_[*P*] for a given differential expression analysis; (2) defined gene sets as groups of these ranked transcripts assigned to the same metabolic pathway; and (3) performed GSEA (fgsea^61^, v.3.14). This set of analyses enabled us to identify differentially expressed metabolic pathways comprised of ≥10 genes over time (1) between treatment groups; (2) between WLZ-response quartiles; or (3) as a function of interacting terms in the linear mixed effect models (treatment group × study week; WLZ-response quartile × study week). Enrichment results were considered to be statistically significant if they exhibited *q* < 0.1 after controlling for false-discovery rate (Benjamini–Hochberg method).

For targeted transcriptional analyses of the CAZymes encoded by *P. copri* MAGs Bg0018 and Bg0019, we used dream^58^ in R with no additional filtering, and the formula above relating transcripts to WLZ response quartile, study week and the interaction of both terms, with the addition of a random effect for participant.

### PCA

PCA was performed on VST-transformed DNA or transcript counts for the 837 MAGs passing the filter described in the ‘Defining the relationship between MAG abundances and WLZ’ section above. The PCA performed on transcript abundances encompassed 27,518 genes expressed by these MAGs at thresholds for levels and prevalence that are described in the ‘Microbial RNA-seq analysis of MAG gene expression’ section above. PCA was performed in R using the ‘prcomp’ function, with each data type centred but not scaled as the dataset was already VST-normalized. The functions ‘get_eigenvalues,’ ‘get_pca_ind’ and ‘get_pca_var’ from the factoextra^62^ (v.1.0.7) package were used to extract (1) the variance explained by each PC; (2) the coordinates for each sample along PCs; and (3) the contributions of each variable to PC1–3. We used the ‘adonis2’ function within the vegan^63^ library (v.2.5-7) to test for the statistical significance of differences in the microbiome (MAGs) or metatranscriptome between the two treatment groups at baseline or over time.

### LC–MS analyses of carbohydrates present in MDCF-2, RUSF, their component ingredients, faecal specimens and culture medium

#### Sample preparation for glycan structure analysis

Frozen samples of MDCF-2, RUSF, their respective ingredients and faecal biospecimens were ground with a mortar and pestle while submerged in liquid nitrogen. A 50 mg aliquot of each homogenized sample was lyophilized to dryness. Lyophilized samples were shipped to the Department of Chemistry at the University of California, Davis. On receipt, the samples were pulverized to a fine powder using 2 mm stainless-steel beads (for foods) or 2 mm glass beads (for faeces). A 10 mg ml^−1^ stock solution of each sample was prepared in Nanopure water. All stock solutions were again bead homogenized, incubated at 100 °C for 1 h, bead homogenized again and stored at −20 °C until further analysis.

#### Monosaccharide composition analysis

Methods were adapted from previous publications^64,65^. For analyses of food ingredients and faecal biospecimens, 10 µl aliquots were withdrawn from homogenized stock solutions and transferred to a 96-well plate containing 2 ml wells. For analyses of monocultures of *P. copri* strains grown in the presence of different purified polysaccharides, microplates were withdrawn from anaerobic chamber at the conclusion of the incubation and centrifuged (5,000*g* for 5 min). The resulting supernatants were removed and immediately frozen at −80 °C.

Each sample was subjected to acid hydrolysis (4 M trifluoroacetic acid for 1 h at 121 °C) followed by addition of 855 µl of ice-cold Nanopure water. Hydrolysed samples, plus an external calibration standard comprising 14 monosaccharides with known concentrations (0.001–100 µg ml^−1^ each) were derivatized with 0.2 M 1-phenyl-3-methyl-5-pyrazolone (PMP) in methanol plus 28% NH_4_OH for 30 min at 70 °C. The derivatized glycosides were fully dried by vacuum centrifugation, reconstituted in Nanopure water (Thermo Fischer Scientific) and excess PMP was extracted with chloroform. A 1 µl aliquot of the aqueous layer was injected into the Agilent 1290 Infinity II ultrahigh-performance liquid chromatography (UHPLC) system, separated using a 2 min isocratic elution on a C18 column (Poroshell HPH, 2.1 × 50 mm, 1.9 μm particle size, Agilent Technologies) and analysed using the Agilent 6495A triple quadrupole mass spectrometer (QqQ-MS) operated in dynamic multiple-reaction-monitoring mode. Monosaccharides in the food and faecal samples were identified and quantified by comparison to the external calibration curve.

#### Glycosidic linkage analysis

Methods were adapted from a previous publication with modifications^66,67^. Under an argon atmosphere, a 5 µl aliquot from each homogenized stock solution of a sample was permethylated in a 200 µl reaction that contained 5 µl saturated NaOH and 40 µl iodomethane in 150 µl of DMSO. Permethylated glycosides were extracted with dichloromethane, and the extract was dried by vacuum centrifugation. The extracted glycosides were processed for acid hydrolysis (4 M trifluoroacetic acid for 2 h at 100 °C) followed by vacuum centrifugation to dryness. The samples were then derivatized with PMP as described above for monosaccharide analysis, followed by another vacuum centrifugation to complete dryness. Methylated monosaccharides were then reconstituted with 100 µl of 70% methanol in water. A 1 µl aliquot of the aqueous layer was injected into the Agilent 1290 Infinity II UHPLC system, separated using a 16 min gradient elution on a C18 column (ZORBAX RRHD Eclipse Plus, 2.1 × 150 mm, 1.8 μm particle size, Agilent Technologies), and analysed using the Agilent 6495A QqQ-MS operated in multiple-reaction-monitoring mode. A standard pool of oligosaccharides and a reference MRM library were used to identify and quantify glycosidic linkages in all of the samples.

#### FITDOG polysaccharide analysis

Methods were adapted from previous publications^17,68^. To separate endogenous oligosaccharides from the background food matrix, polysaccharides were precipitated with 80% aqueous ethanol. Dried precipitates were reconstituted in water to 10 mg ml^−1^ and then homogenized. The Fenton’s initiation toward defined oligosaccharide groups (FITDOG) reaction was performed using a 100 μl aliquot of the 10 mg ml^−1^ resuspended food pellet and 900 μl of reaction buffer (44 mM sodium acetate, 1.5% H_2_O_2_, 73 µM Fe_2_(SO_4_)_3_(H_2_O)_5_). The reaction mixture was incubated at 100 °C for 45 min, quenched with 500 μl 2 M NaOH and then neutralized with 61 μl of glacial acetic acid. The resulting oligosaccharides were then reduced to their corresponding alditols with sodium borohydride (NaBH_4_) to prevent anomerization during chromatographic separation. For the reduction of oligosaccharides, a 400 μl aliquot of the reaction mixture was incubated with 400 μl 1 M NaBH_4_ at 65 °C for 60 min. Oligosaccharide products were then enriched using C18 and porous graphitized carbon (PGC) 96-well solid-phase extraction plates. For the C18 enrichment, cartridges were primed with two washes with 250 μl acetonitrile and then five washes with 250 μl water washes before loading the reduced sample. The cartridge effluent was collected and processed for subsequent PGC clean-up. PGC cartridges were primed with 400 μl water, 400 μl 80% acetonitrile/0.1% (v/v) trifluoroacetic acid, and then 400 μl water before loading the C18 effluent. Washing was performed with 8 × 400 μl water, and the oligosaccharides were eluted with 40% acetonitrile/0.05% (v/v) trifluoroacetic acid and then dried using a vacuum centrifugal dryer. Oligosaccharides were reconstituted with 100 μl Nanopure water and a 10 μl aliquot was injected into the HPLC–Q-TOF instrument. Separation was performed using the Agilent 1260 Infinity II HPLC system with a PGC column (Hypercarb, 1 × 150 mm, 5 μm particle size, Thermo Fisher Scientific) coupled to the Agilent 6530 Accurate-Mass Q-TOF mass spectrometer. Specific HPLC, electrospray source and MS acquisition parameters are described in greater detail in previous publications^17,68^. Oligosaccharide identification was based on MS/MS fragmentation and retention time compared to reacted polysaccharide standards (amylose, cellulose, mannan, galactan, linear arabinan and xylan). Food polysaccharides were quantified using an external calibration curve that included the three most abundant oligosaccharides from each parent polysaccharide as the quantifier species.

#### Statistical analysis of carbohydrate composition

We analysed the abundances of glycosidic linkages over time and between WLZ-response quartiles using linear mixed-effects models (lme4^56^, lmerTest^57^ packages in R) of the following form:

$$\begin{array}{l}{{\rm{linkage}}}_{i} \sim {\beta }_{1}({\rm{WLZ}} \mbox{-} {\rm{response\; quartile}})+{\beta }_{2}({\rm{study}}\;{\rm{week}})\\ \,\,\,\,+\,{\beta }_{3}({\rm{WLZ}} \mbox{-} {\rm{response\; quartile}}\times {\rm{study}}\;{\rm{week}})+(1| {\rm{PID}})\end{array}$$

Linkages displaying a significant interaction (*q* < 0.05) between WLZ-response quartile and study week (*β*_3_ coefficient) were identified.

### Culturing *P. copri* from faecal samples and genome sequencing

Faecal samples, obtained from our previously reported studies of Bangladeshi children living in Mirpur^3,4^, were first screened on the basis of the abundances of *P. copri* V4-16S rDNA amplicon sequence variants and/or *P. copri* MAGs. Five samples from our previous pilot MDCF study^3^, plus an additional 32 samples from the larger randomized controlled clinical trial (prioritized on the basis of participants’ membership in the upper quartile of WLZ response to MDCF-2 treatment) were selected for this culturing effort.

A frozen aliquot (~0.1 g) of each selected faecal sample was weighed. All of the subsequent steps were performed under anaerobic conditions (atmosphere; 75% N_2_, 20% CO_2_, 5% H_2_) in a Coy chamber (Coy Laboratory Products). For the faecal samples that yielded strains BgD5_2, BgF5_2 and BgG5_1, aliquots were resuspended directly in 100 µl of phosphate-buffered saline (PBS) containing 0.5% (w/v) l-cysteine. All of the other samples were clarified as described previously^3^. A 100 µl aliquot of each resuspended or clarified sample was serially diluted in PBS containing 0.5% (w/v) l-cysteine and plated onto Laked sheep blood–kanamycin–vancomycin agar plates (Hardy Diagnostics, A60). *Prevotella* spp. produce a pigment known to fluoresce brick red when exposed to ultraviolet light. Thus, colonies grown from serial dilutions were screened for this phenotype, picked and struck onto brain–heart infusion (BHI, Difco, 241830) agar plates containing 10% horse blood. Individual isolated colonies were picked into liquid Wilkins–Chalgren medium (Oxoid, CM0643), grown overnight at 37 °C and then mixed 1:1 with prereduced 30% glycerol (in PBS plus 0.5% (w/v) l-cysteine) in 1.8 ml glass vials (E-Z vials, Wheaton). Vials were closed by crimping on a cover containing a PTFE/grey butyl liner (Wheaton). Stocks were preserved at −80 °C for later use. An additional aliquot of each culture was processed for full-length 16S rDNA sequencing (universal primers 8F and 1391R) to provide an initial taxonomic identification. This effort yielded a total of 108 isolates assigned to *Prevotella* spp., including 86 classified as *P. copri*.

Each *P. copri* isolate was retrieved from storage, cultured in Wilkins–Chalgren medium and processed for whole-genome sequencing. Freezer stocks of each bacterial isolate were inoculated into Wilkins–Chalgren medium and grown at 37 °C under anaerobic conditions without shaking until reaching late-log phase. A 6 ml aliquot of each culture was withdrawn from the anaerobic chamber and centrifuged at 5,000*g* for 5 min. The resulting cell pellet (10–50 mg) was transferred to a 2 ml cryotube and DNA was extracted by bead beating (Biospec Minibeadbeater-96) for 1 min with a 3.97 mm steel ball and 250 μl of 0.1 mm zirconia/silica beads in 500 μl of 25:24:1 phenol:chloroform:isoamyl alcohol solution, 210 μl of 20% SDS and 500 μl of 2× buffer A (200 mM NaCl, 200 mM Trizma base, 20 mM EDTA)^69^. The resulting mixtures were centrifuged at 3,220*g* for 4 min at 22 °C. A 420 μl aliquot of the resulting aqueous phase was transferred to a deep 96-well plate and purified using the QIAquick 96-well PCR purification kit (Qiagen). DNA was quantified (Quant-iT dsDNA broad range kit; Invitrogen) and the fragment-size distribution was measured (TapeStation using a genomic DNA ScreenTape (Agilent)).

Purified DNA was prepared for long-read sequencing using the SMRTbell Express Template Prep Kit 2.0 from Pacific Biosciences (Pacific Biosciences; PacBio); we followed the manufacturer’s instructions for creating HiFi Libraries from low DNA input, with adjustments made to accommodate a 96-well plate configuration^69^. The DNA concentration and fragment-size distribution of resulting libraries were evaluated (genomic DNA ScreenTape; TapeStation instrument) and the libraries were pooled at equimolar concentrations after normalizing for expected genome size. Pooled libraries were sequenced on the Sequel long-read DNA sequencer (PacBio) using the Sequel Binding Kit 3.0 and Sequencing Primer v4, with 24 h of data collection. The samples were demultiplexed, and Q20 circular consensus sequencing reads were generated using the Cromwell workflow from PacBio. The Flye^70^ assembler (v.2.8.1) was used to assemble the genomes, with the HiFi-error set to 0.003, min-overlap set at 2000 and other options kept as the default. Genome quality was evaluated using CheckM^40^ (v.1.1.3), and genomes were annotated using prokka^44^ (v.1.14.6).

### Constructing a marker-gene-based phylogeny for *P. copri* MAGs and cultured *P. copri* isolates

We used CheckM^40^ (v.1.1.3) to extract and align the amino acid sequences of 43 single-copy marker genes in each of the 11 *P. copri* MAGs, each of the 6 cultured *P. copri* strains, plus the type strain of *Bacteroides thetaiotaomicron* VPI-5482 (accession number: 226186.12). Concatenated, aligned marker gene sequences were analysed (fasttree^71^; v.2.1.10) using the Jones–Taylor–Thornton model and ‘CAT’ evolution rate to create a phylogenetic tree, which was subsequently rescaled using the ‘Gamma20’ optimization. The resulting tree was rooted to the *B. thetaiotaomicron* genome (‘ape’, v.5.6-2^72^) before extracting the phylogenetic distances between MAGs and isolates. The tree was visualized using ggtree^73^ (v.3.2.1).

### Subsystem-based annotation and prediction of functional capabilities (inferred metabolic phenotypes) of MAGs and cultured *P. copri* strains

MAG and isolate genes were assigned functions, and metabolic pathways were reconstructed using a combination of (1) public-domain tools for sequence alignment and clustering; (2) custom scripts to process the results of sequence alignments (for example, for domain annotation in multifunctional proteins); and (3) a reference collection of 2,856 human gut bacterial genomes for which we have reconstructed and manually curated metabolic pathways related to 98 distinct metabolites and 106 metabolic phenotypes^16^. These annotations are captured in the mcSEED database, a microbial community-centred adaptation of the SEED genomic platform^14,15^, featuring subsystem-based annotation and pathway reconstruction applied to representative human gut bacterial genomes that were initially automatically annotated by RAST or downloaded from the PATRIC (recently renamed Bacterial and Viral Bioinformatic Resource Center, BV-BRC) database^74^. Each mcSEED subsystem includes a set of functional roles (for example, enzymes, transporters, transcriptional regulators) that contribute to the prediction of functional metabolic pathways and pathway variants^75^ that are involved in the utilization and catabolism of carbohydrates and amino acids, biosynthesis of vitamins/cofactors and amino acids, and generation of fermentation end-products such as short-chain fatty acids. A complete list of MAG genes comprising these pathways, their abbreviations and functions is provided in Supplementary Table 5 (annotations are based on the January 2021 version of the mcSEED database).

Our annotation workflow is shown in Supplementary Fig. 1. In brief, we constructed a reference database containing 995,591 functionally annotated proteins comprising the entire set of curated metabolic subsystems from the 2,856 reference genomes plus an additional 2,988,751 proteins (outgroup is not included in these metabolic subsystems), clustered at 90% amino acid identity (‘cluster’ command, MMSeqs^76^, v.1-c7a89). We aligned the predicted protein sequences from the set of 1,000 high-quality MAGs against this reference protein database (DIAMOND^77^, v.2.0.0). To account for any influence of MAG fragmentation on metabolic reconstruction, we also identified gene fragments using prodigal^78^ (v.2.6.3) and annotated them in parallel. We implemented the following method to account for instances of multidomain structure that require multiple annotations. For each MAG query protein, we used the top 50 hits based on the bitscore, clustered the start and end position coordinates of the corresponding alignments (DBSCAN function, Scikit-learn^79,80^, v.0.22.1), used the centre of each clustered start and end position as potential domain boundary coordinates, and split query proteins into domains with database hits attributed to the corresponding domains. Next, for each domain of ≥35 amino acids, we used Gaussian kernel density modelling (KernelDensity function, neighbours module, Scikit-learn^80^) of the sequence identity distribution of each set of hits to that domain. A highest local minimum (argrelextrema function, signal module, Scikit-learn^80^) was used as a threshold to remove low-confidence hits. Finally, functional annotations were applied from the reference database to each query protein or domain by majority rule within each set of high-scoring, domain-specific reference hits. High-identity hits to proteins from the outgroup of the reference database were used as criteria to vote against applying annotation to each query. This procedure yielded a set of 199,334 annotated MAG proteins, representing 1,308 unique protein products across a set of 80 mcSEED subsystems (Supplementary Table 5).

#### Phenotype prediction strategies

We integrated the results of gene-level functional annotation into in silico predictions of the presence or absence (denoted as binary: ‘1’ for presence or ‘0’ for absence) of 106 functional metabolic pathways (Supplementary Table 7) using a semi-automated process based on a combination of the following three approaches:

#### Pathway-rules-based phenotype predictions

This approach uses explicit logic-based ‘pathway rules’ to assign binary phenotypes. These rules combine (1) expert curators’ knowledge regarding the gene composition of various metabolic pathway variants contained in the mcSEED database with (2) a decision tree method to identify patterns of gene representation in reference genomes corresponding to an intact functional pathway variant (and a respective binary phenotype value denoted as ‘1’). A total of 106 functional pathway-specific decision trees was generated (Rpart^81^, v.4.1.15), where the presence or absence of a particular phenotype was the response variable, and the presence or absence of functional roles (encoded by genes) in each reference pathway were predictor variables. The resulting pathway rules were formally encoded into a custom R script that enabled us to process MAG gene data and assign values (1 or 0) for each of the 106 functional metabolic pathways.

#### Machine-learning-based phenotype predictions

We compared >30 ml methods (Caret^82^, v.6.0.86), using a leave-one-out cross-validation approach in which we removed a single reference genome from the set of 2,856 reference genomes, trained machine-learning models on the remaining genomes, then applied the models to the ‘test’ genome to predict phenotypes. This procedure was then repeated for each genome and each metabolic phenotype. The results of this analysis identified random forest as the best-performing method (that is, it produced the greatest number of correctly predicted phenotypes in our reference training dataset). We then built random-forest models for each phenotype based on the reference dataset, optimized model parameters using a grid search and used these models to predict binary (1/0) values for the same set of 106 phenotypes for all MAGs.

#### Neighbour-group-based phenotype predictions

This approach identifies reference bacteria that are closely related to the MAGs in this study and uses these high-quality reference genomes for phenotype predictions that are robust to variation in MAG quality. Examination of groups of closely related reference organisms suggested that close phylogenetic neighbour genomes tend to either possess or lack an entire pathway variant, whereas more distant neighbours (such as other neighbour groups) often carry more divergent pathway variants that specify the same phenotype. We used this observation to develop heuristics that minimize false-negative phenotype assignments emerging from the other two prediction strategies. We compiled a set of neighbour groups comprising MAGs and closely related reference genomes (Mash/MinHash^83^ distance ≤  0.1, corresponding to ANI ≥ 90%). At this similarity threshold, we assigned 640 out of the 1,000 MAGs from this study to neighbour groups containing as few as four to more than 100 members. Within each neighbour group and for each metabolic pathway, we tentatively assigned a binary phenotype value for a given MAG based on the neighbour-group genome with the closest matching gene annotation pattern (based on Hamming distance), even if some of the genes were absent in the query MAG. We limited comparisons to genes required for the function of each respective pathway.

#### Consensus phenotype predictions

We established a procedure to reconcile inconsistent phenotype predictions between the three strategies described above, based on observing discordant gene patterns and/or discordant predicted phenotypes within a given group of neighbour genomes. In the rare case of an irreconcilable disagreement between the prediction methods, assignment of a consensus phenotype defaulted to that produced by the machine-learning method. We assigned consensus confidence scores to each prediction on the basis of the degree of concordance between the three techniques and our confidence in the accuracy of each (Supplementary Discussion and Supplementary Table 7a). The complete phenotype prediction process was validated using the 2,856 reference genomes in the mcSEED database, their functionally annotated genes and the accompanying patterns of presence/absence of functional metabolic pathways (curator-inferred binary phenotypes). The consensus phenotype predictions were combined into a binary phenotype matrix (BPM) containing 1,000 MAGs and 106 phenotypes (Supplementary Fig. 1, Supplementary Table 7a).

#### Gene annotation and phenotype prediction for *Bifidobacterium*-specific carbohydrate-utilization pathways

We adapted the annotation pipeline described above (Supplementary Fig. 1) to obtain functional annotations of genes comprising 25 additional carbohydrate-utilization pathways for a set of 34 *Bifidobacterium* MAGs, followed by inference of their respective binary phenotypes. As input data for this set of *Bifidobacterium*-specific phenotypes, we curated a set of 14 metabolic subsystems in 387 reference human-gut-derived *Bifidobacterium* genomes using the mcSEED framework. The reconstructed metabolic pathways and a corresponding BPM for reference *Bifidobacterium* genomes were used to predict carbohydrate-utilization phenotypes in the 34 *Bifidobacterium* MAGs. Finally, the automatically generated BPM was further manually curated to account for the variability of certain pathways in this taxonomically restricted set of predictions.

#### Applying enrichment analyses to predicted MAG phenotypes

Not all successfully annotated MAG genes were components of an intact functional pathway. To enable inferred phenotype-based analysis, we filtered gene annotations to those that were part of a complete functional pathway (with a respective binary phenotype value denoted as ‘1’). This filter resulted in a list of 208,246 genes used for microbiome and metatranscriptome phenotype enrichment analyses.

### Annotation of CAZymes and PULs in *P. copri* MAGs and cultured *P. copri* strains

CAZymes were annotated according to the CAZy classification scheme^84^. Amino acid sequences from MAGs and isolate genomes were analysed using a bioinformatics workflow that performs homology searches against the CAZy database^85^ and specifically accommodates the modular structure of CAZymes (which often carry a variable number of ancillary modules in addition to their catalytic domain). Details of this workflow and its application are provided in a previous publication^86^.

PULs were identified/predicted by combining information from marker genes (SusC/SusD pairs), operon structure and CAZyme annotation information, as previously described^24^. The experimentally validated substrate specificities of CAZyme homologues contained in each PUL were used to infer the carbohydrate substrate(s) of each PUL.

We investigated the conservation of PULs within WLZ-associated *P. copri* MAGs and, subsequently, between these MAGs and all other *P. copri* MAGs and cultured *P. copri* isolate genomes from this study. As no automated method has been described to perform such a pan-genome PUL comparison, we proceeded manually through the following steps. First, we predicted the PUL repertoire of Bg0018 and Bg0019 using PULDB^25^. Next, using Bg0019 as a reference, we selected PULs in Bg0018 and in other genomes for additional analysis if they displayed the same gene components in the same genomic order in both genomes. To do so, we conducted pairwise searches (BLASTp^51^) to identify homologous components (for example, SusC, SusD, transcriptional regulators, genes encoding CAZymes) of PULs in other *P. copri* genomes. The level of conservation for each PUL across the set of MAGs was categorized as (1) conserved (pairwise comparisons of ORFs satisfy the requirements that their protein products have >90% amino acid identity and that the ORFs comprising the PULs being compared are organized in an identical way within the respective genomes); (2) structurally distinct (a given PUL is present in the genomes being compared but one or more CAZymes or one or both SusC/SusD proteins are missing or fragmented in a way that is likely to impact their function, or where extra PUL ORFs are present); or (3) not conserved/absent (PULs present in the respective genomes but with mutations that are likely to completely compromise function or where no PUL identified).

To relate the pattern of PUL conservation in *P. copri* MAGs to the WLZ response of study participants, we re-encoded each cell in the matrix shown in Fig. 4a as ‘1’ for conserved PULs, ‘0.5’ for structurally distinct PULs and ‘0’ for not conserved/absent PULs. We imported this matrix into R, calculated a Euclidean distance between the PUL conservation pattern of Bg0019 and each additional MAG, then hierarchically clustered these patterns to generate a tree. Finally, we used Pearson correlation to relate the distance between the patterns of PUL conservation in Bg0019 and each additional *P. copri* MAG to the WLZ association (*β*_1_ coefficient) of each MAG (Fig. 4b).

### In vitro screening of glycan substrate specificity

We selected a variety of growth substrates representing putative glycan components of MDCF-2 plus glucose (Sigma-Aldrich, G8270) as a positive control and chondroitin sulfate (Thermo Fisher Scientific, J66156.06) as a negative control. Growth substrates included arabinan, arabinoxylan, β-glucan, galactan, galactomannan, rhamnogalacturonan, xyloglucan and xylan (all obtained from Neogen Megazyme; Supplementary Table 16a). A 2% (w/v) solution of each polysaccharide was prepared by mixing the polysaccharide with autoclaved, filter-purified water as recommended by the supplier (see Supplementary Table 16a for additional details of the preparation of each solution). Each solubilized substrate was allowed to equilibrate in the anaerobic growth chamber for around 5 days before use. Sterility was checked by (1) plating 10 µl aliquots of each polysaccharide preparation onto BHI + 10% horse blood agar and (2) preparing 1:1 mixtures of preparation in *P. copri* defined medium (PCDM). This growth assay medium was selected because (1) it was fully defined, enabling supplementation with specific carbon sources and (2) it successfully supported the growth of *P. copri* strains in preliminary test cultures containing 5% glucose as the sole carbon source. PCDM was prepared according to the published recipe^87^ with slight modifications (1 ml of a solution containing 1.9 mM haematin (H3281, Sigma-Aldrich) and 0.2 M l-histidine (Sigma-Aldrich, H6034) was added to each litre of the medium). Plates and broth were incubated for ≥3 days before checking for growth. The galactan and rhamnogalacturonan solutions displayed contamination. Thus, a fresh 2% (w/v) solution of each of these polysaccharides was prepared and autoclaved at 121 °C for 20 min at 15 psi before transferring them to the anaerobic chamber and confirming their sterility as described above. Autoclaved stocks were again checked for sterility before use.

We selected five *P. copri* isolates, representing a diversity of PUL conservation profiles, mcSEED BPMs and phylogenetic assignments for in vitro growth assays. Freezer stocks of each isolate were plated (~200 µl) onto BHI + 10% horse blood and grown for 48 h at 37 °C. Isolated colonies were picked into 3 ml Wilkins–Chalgren medium and grown for 15 h at 37 °C. Each culture was then diluted 1:100 into fresh Wilkins–Chalgren medium and grown for an additional 6 h under the same anaerobic conditions at 37 °C to bring each culture into a similar phase of growth. The OD_600_ was measured for each culture (Genesys 10S UV-Vis, Thermo Fisher Scientific) and the OD_600_ was standardized to 0.02 by dilution into a final volume of 1 ml of PCDM with no added carbon source in a 96 × 1.3 ml deep-well plate (Nunc, Thermo Fisher Scientific); this procedure enables us to have a uniform inoculum for all test isolates. A total of 9 ml of a 1:1 mixture of 2× PCDM containing 2% (w/v) glycan or control substrate was prepared in 50 ml polypropylene tubes (Corning) yielding 11 tubes of medium, each with an individual carbon source. These media plus the OD-standardized cultures were sealed (Alumaseal, Excel Scientific) and transferred to an anaerobic chamber containing a BioTek Precision XS liquid-handling robot. The robot was used to aliquot 180 μl of each type of medium into corresponding columns of three 96-well flat-bottom tissue culture plates with a well capacity of 340 µl (TPP, Sigma-Aldrich). A separate procedure on the Precision XS robot was used to mix and aliquot 20 µl of each OD_600_-standardized culture into corresponding rows of each assay plate; one row was reserved as a no-inoculation control. Assay plates were then sealed with optically clear films (Axygen, UC-500) and placed into the input stack of a BioTek BioStack 4 microplate stacker configured to load a BioTek Eon microplate spectrophotometer. Before initiating the experiment, two heated catalyst boxes were used to warm the chamber to 37 °C. The microplate stacker and reader were used to perform 1 week of continuous monitoring, with OD_600_ readings obtained every 15 min. Each assay plate was agitated for 5 s before each OD reading. At the conclusion of the experiment, data were exported in tabular form and analysed in R using custom scripts to determine maximal growth rate and OD_600_ for each replicate of each culture in each glycan. Extracted curve parameters were normalized to growth rates determined in PCDM containing 5% glucose as the sole carbon source.

### Biological materials

Faecal specimens collected from Bangladeshi children are the property of icddr,b. Material transfer agreements exist between icddr,b and Washington University in St Louis for the use of these samples. Requests for materials should be made to J.I.G and T.A.

### Statistics and reproducibility

Statistical analyses were conducted using the approaches described in the Methods and the figure legends. Sample sizes and replicates are indicated along with each statistical test. All relevant statistical tests were two-tailed unless otherwise specified. All measurements were collected from distinct samples. Technical replicates were not collected and analysed unless otherwise noted.

### Reporting summary

Further information on research design is available in the Nature Portfolio Reporting Summary linked to this article.

## Online content

Any methods, additional references, Nature Portfolio reporting summaries, source data, extended data, supplementary information, acknowledgements, peer review information; details of author contributions and competing interests; and statements of data and code availability are available at 10.1038/s41586-023-06838-3.

### Supplementary information

Supplementary Information Supplementary Discussion and Supplementary Figs. 1–5.

Reporting Summary

Supplementary Tables 1–5 Supplementary Tables 1–5.

Supplementary Table 6 Supplementary Table 6.

Supplementary Tables 7–18 Supplementary Tables 7–18.

## Data Availability

Shotgun DNA sequencing and microbial RNA-seq datasets generated from faecal samples, plus annotated *P. copri* isolate genome sequences are available at the European Nucleotide Archive (PRJEB45356). These data were analysed to generate Figs. 1, 3 and 4, Extended Data Figs. 1, 2, 4, 5, 7, 8 and 10 and Supplementary Figs. 4 and 5. Anthropometry data from a previous publication^4^ were used in combination with data from the current study to generate Figs. 1b and 4b. The mcSEED database (10.5281/zenodo.10041395) that was used to predict the presence or absence of metabolic pathways shown in Fig. 3, Extended Data Fig. 2 and Supplementary Figs. 1, 4 and 5 was described in a previous publication^16^. The CAZy^84,85^ and PULdb^25^ databases were used to identify and analyse genes encoding carbohydrate-active enzymes and PULs in Figs. 4 and 5 and Extended Data Figs. 5 and 7–9. LC–MS datasets of monosaccharide, glycoside linkage and polysaccharide data are deposited in GlycoPOST (GPST000244) and were used to generate Figs. 2 and 5 and Extended Data Figs. 3, 6 and 7. All other relevant and/or supporting data are available in the Supplementary Information.

## Code availability

Code detailing the steps in the MAG assembly workflow and analyses of microbial RNA-seq and glycan datasets are available at GitLab (https://gitlab.com/hibberdm/hibberd_webber_et_al_mdcf_poc_mags) and have been accessioned at Zenodo (10.5281/zenodo.8000098). Code for annotation of bacterial genes and prediction of metabolic pathway presence/absence is available at GitHub (https://github.com/rodionovdima/PhenotypePredictor) and has been accessioned at Zenodo (10.5281/zenodo.10049439).
