# Supplementary material for: Bioactive glycans in a microbiome-directed food for children with malnutrition
Source: Nature. 2023 Dec 13;625(7993):157–65. doi: 10.1038/s41586-023-06838-3 (PMC10764277; doi:10.1038/s41586-023-06838-3)
Supplement: Supplementary file 1 — Supplementary Discussion and Supplementary Figs. 1–5. [file 41586_2023_6838_MOESM1_ESM.docx]

**Bioactive glycans in a microbiome-directed food for malnourished children**

Matthew C. Hibberd^1,2,3,†^, Daniel M. Webber^1,2,3,†^, Dmitry A. Rodionov^4^, Suzanne Henrissat^1,2,5^, Robert Y. Chen^1,2^, Cyrus Zhou^1,2^, Hannah M. Lynn^1,2^, Yi Wang^1,2^, Hao-Wei Chang^1,2^, Evan M. Lee^1,2^, Janaki Lelwala-Guruge^1,2^, Marat D. Kazanov^6^, Aleksandr A. Arzamasov^4^, Semen A. Leyn^4^, Vincent Lombard^5^, Nicolas Terrapon^5^, Bernard Henrissat^7,8^, Juan J. Castillo^9^, Garret Couture^9^, Nikita P. Bacalzo, Jr.^9^, Ye Chen^1,2,9^, Carlito B. Lebrilla^9^, Ishita Mostafa^10^, Subhasish Das^10^, Mustafa Mahfuz^10^, Michael J. Barratt^1,2,3^, Andrei L. Osterman^4^, Tahmeed Ahmed^10^ and Jeffrey I. Gordon^1,2,3,*^

^†^Contributed equally

^1^Edison Family Center for Genome Sciences and Systems Biology, Washington University School of Medicine, St. Louis, MO 63110 USA

^2^Center for Gut Microbiome and Nutrition Research, Washington University School of Medicine, St. Louis, MO 63110 USA

^3^Department of Pathology and Immunology, Washington University School of Medicine, St. Louis, MO 63110 USA

^4^Infectious and Inflammatory Disease Center, Sanford Burnham Prebys Medical Discovery Institute, La Jolla, CA 92037 USA

^5^Architecture et Fonction des Macromolécules Biologiques, CNRS, Aix-Marseille University, F-13288, Marseille, France

^6^Faculty of Engineering and Natural Sciences, Sabanci University, Istanbul, Turkey, 34956

^7^Department of Biotechnology and Biomedicine (DTU Bioengineering), Technical University of Denmark, DK-2800 Kgs. Lyngby, Denmark

^8^Department of Biological Sciences, King Abdulaziz University, Jeddah, Saudi Arabia

^9^Department of Chemistry, University of California, Davis, CA 95616, USA

^10^International Centre for Diarrhoeal Disease Research, Bangladesh (icddr,b), Dhaka 1212, Bangladesh

*Address correspondence to: jgordon@wustl.edu

**SUPPLEMENTARY INFORMATION**

**Supplementary Discussion**

**Performance of short-read sequencing only versus hybrid (long- and short-read) MAG assembly**

We explored the impact of the addition of long-read sequencing data on various quality characteristics of MAGs assembled from data collected from the 0- and 3-month time points from all upper WLZ quartile responders (n=15) in the MDCF-2 treatment group. In our final set of high-quality, dereplicated MAGs, 918 MAGs represented contigs assembled from short-read only data, while 82 were derived from hybrid short and long-read assemblies (**Supplementary Table 1**). Although the mean quality characteristics of MAGs from each assembly type did not differ in completeness (determined by marker gene analysis) or total length, MAGs derived from hybrid assemblies displayed a significantly lower rate of contamination, fewer contigs, and greater N50 (see **Extended Data Fig. 1b** for statistical analyses).

**Comparing MAG assembly accuracy and quantitation using a pseudoalignment and expectation maximization approach**

MAG assembly algorithms that synthesize both contig sequence characteristics and contig abundance to assemble MAGs (*e.g*., MaxBin2^35^, MetaBAT2^36^) require accurate contig quantitation. Alignment-free quantitation approaches (*e.g.*, kallisto) have demonstrated superior speed and accuracy compared to read- mapping-based quantitation in the context of metagenomic analyses where read-mapping ambiguity is common^45^.

We investigated the utility of kallisto-based quantitation for (i) contigs, prior to MAG assembly, and (ii) MAGs themselves after assembly and curation. For this analysis, we employed a ‘mouse gut metagenome toy dataset’ from CAMI II that included 64 ‘mock fecal samples’; these mock samples were produced using sequencing data from 791 publicly available bacterial genomes (representing 549 species) and genomic abundances that mirrored bacterial 16S rRNA gene profiles of 64 actual mouse fecal biospecimens^88^. We utilized three components of this reference dataset for our analyses: (i) simulated sequencing data (1.67×10^7^ Illumina paired-end 150 nt reads) from each of the 64 mock fecal samples, (ii) anonymized reference contigs from the 791 reference genomes, and (iii) reference abundances of contigs/genomes in each fecal sample.

First, we investigated the effect of kallisto quantification of contigs on the fidelity of MAG assembly. To do so, we quantified the reference contigs using either kallisto or bowtie2 and the short-read simulated Illumina data. Next, we assembled MAGs using MaxBin2^35^, MetaBAT2^36^ and CONCOCT^89^ with data from either kallisto or bowtie2 contig quantitation as input. The output of each MAG assembly method for each sample was combined using DAS Tool^37^. Finally, each MAG set was compared against 791 intact reference genomes using AMBER^90^ (**Extended Data Fig.** **10a,b**). MAGs generated using kallisto contig quantification and DAS Tool dereplication were more complete (*P*=6.4×10^-14^; Wilcoxon test) and less contaminated (*P*<2.2×10^-16^; Wilcoxon test) than those generated using bowtie2 (**Extended Data Fig. 10c**). Additionally, a significantly greater number of MAGs (*P*<0.05; Fisher's exact test) were detected using kallisto contig quantitation (**Extended Data Fig. 10d**).

Next, we employed the same simulated dataset to test the accuracy of kallisto-based MAG quantitation. We mapped the short-read data for each of the 64 fecal samples to the set of 791 reference genomes using kallisto and bowtie2. We then correlated the abundance profiles generated by each quantitation method to the ‘true’ abundance profile for each sample. The correlations between true genome abundances and kallisto genome abundances were stronger than those calculated using bowtie (mean Pearson’s *r*^2^= 0.99 for kallisto versus *r*^2^= 0.97 for bowtie; *P*<2.2×10^-16^, Wilcoxon test comparing each distribution of correlation coefficients).

We determined the false positive and false negative rate of MAG detection across all samples. Notably, kallisto quantitation resulted in more false positive abundances across the 64 mock fecal samples [300.2±50.1 versus 69.3±28.4 for bowtie2, respectively (mean±SD); *P*< 2.2x10^-16^, Wilcoxon test] while bowtie2 quantitation resulted in more false negative abundances [0.09±0.42 versus 17.2±26.1 (mean±SD), respectively; *P*<2.2×10^-16^, Wilcoxon test]. Importantly, analysis of the average values of false positive abundance generated using kallisto suggested that a low abundance filter would significantly reduce the false positive rate. For example, applying a filter to this dataset that required >5 TPM for a MAG to be designated as ‘detected’ resulted in a false positive rate significantly lower than that of bowtie2 (*P*=0.02, Wilcoxon test).

The greater number of high quality (less contaminated and more complete) MAGs assembled using kallisto quantitation, plus the increased accuracy of MAG quantitation using this method, led us to employ kallisto for all quantitation tasks in the MAG analysis workflow described in the current study.

**Analysis of consistency in MAG functional metabolic pathway annotation**

A global comparison of binary phenotype assignments derived using the Pathway Rules (PR), Machine Learning (ML), and Neighbor Group (NG) approaches described in *Methods* revealed a remarkably low frequency of inconsistencies: in a subset of 640 MAGs where all three methods could be applied, only 4.5% of NG-based phenotype assignments were inconsistent between one or more other methods. These inconsistencies reflect different biases associated with each approach. The NG-based approach exhibits limited performance for small (<5-member) NGs with underrepresented local diversity of gene patterns. Alternatively, PR/ML-based methods appear to be less robust with respect to genome incompleteness in MAGs, resulting in omission (absence) of genes essential for the function of a pathway and, more generally, for pathways with less than three essential genes. Our consensus approach (*Methods*) resolved 70% of observed inconsistencies toward PR/ML-based assignments. In the remaining cases, a consensus phenotype was assigned in favor of the NG-based method. The overall level of inconsistencies between PR- and ML-based phenotype assignments across the entire set of 199,334 assignments in the 1,000 MAGs was much lower (<0.7%). A detailed investigation of selected cases showed that, in general, the ML-based method yielded higher accuracy phenotype assignments. Therefore, in rare cases of irreconcilable disagreement between these two methods in the set of 360 MAGs without NGs, the semi-automated assignment of the consensus phenotype was made in favor of the ML-based approach.

**Non-carbohydrate related differentially expressed transcripts in upper versus lower WLZ quartile responders**

Transcripts expressed at greater levels in upper WLZ response quartile participants (β_1_ WLZ quartile term) were also enriched for pathways involved in biosynthesis of vitamin B3 and B9 and the essential amino acids tryptophan, lysine, histidine and leucine (**Supplementary** **Table 14b**). Leading-edge analysis revealed that *P. copri* Bg0018 and Bg0019 were major contributors to increased expression of transcripts involved in the biosynthesis of vitamins B3 and B9, plus the essential amino acids tryptophan, histidine, leucine and lysine, among the upper quartile responders, but contributed minimally (two transcripts assigned to the arginine biosynthetic pathway) to enrichment of functional pathways among the lower WLZ quartile responders (**Supplementary** **Table 14b**).

**Supplementary References**

88. Meyer, F. et al., Tutorial: Assessing metagenomics software with the CAMI benchmarking toolkit. Nature Protocols, 16, 1785-1801 (2021).

89. Alneberg, J. et al., Binning metagenomic contigs by coverage and composition. Nature Methods 11, 1144–1146 (2014).

90. Meyer, F. et al., AMBER: Assessment of metagenome BinnERs. GigaScience 7, giy069 (2018).

**Supplementary Figures**

**
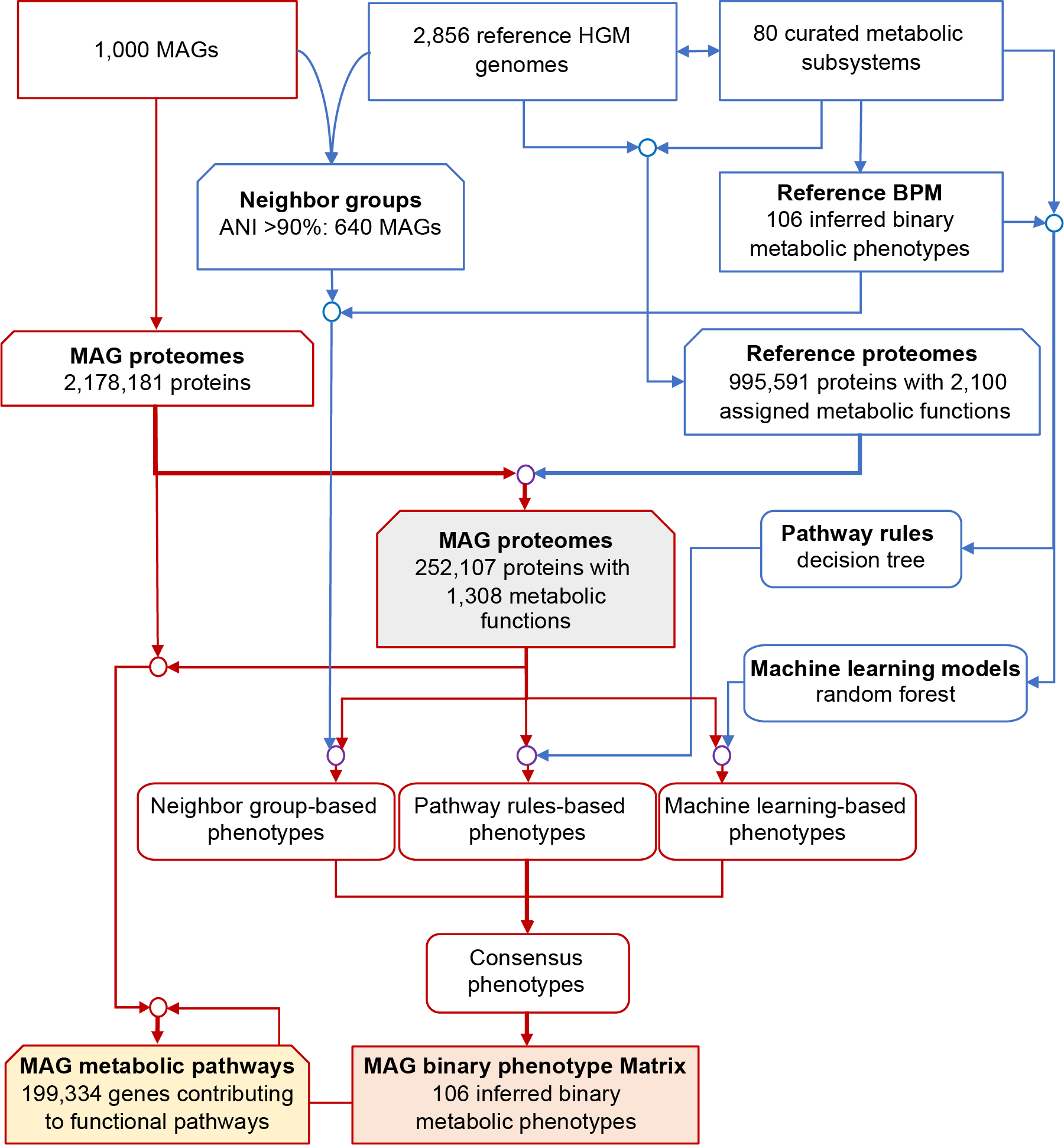
**

Supplementary Fig. 1. Bioinformatics pipeline for subsystems-based annotation and prediction of functional capabilities (metabolic phenotypes) of MAGs. The flow diagram shows the input data (2,856 reference genomes, 80 curated metabolic subsystems and 1,000 target MAGs) and the main computational steps performed. The pipeline produces two major outputs: (i) a complete set of functionally annotated proteins contributing to 80 reconstructed metabolic subsystems identified in the collection of 1,000 MAGs (annotation results are detailed in Supplementary Table 5 and Supplementary Table 6); and (ii) a Binary Phenotype Matrix (BPM) reflecting the inferred presence or absence of 106 functional metabolic pathways in each of the 1,000 MAGs shown in Supplementary Table 7a.


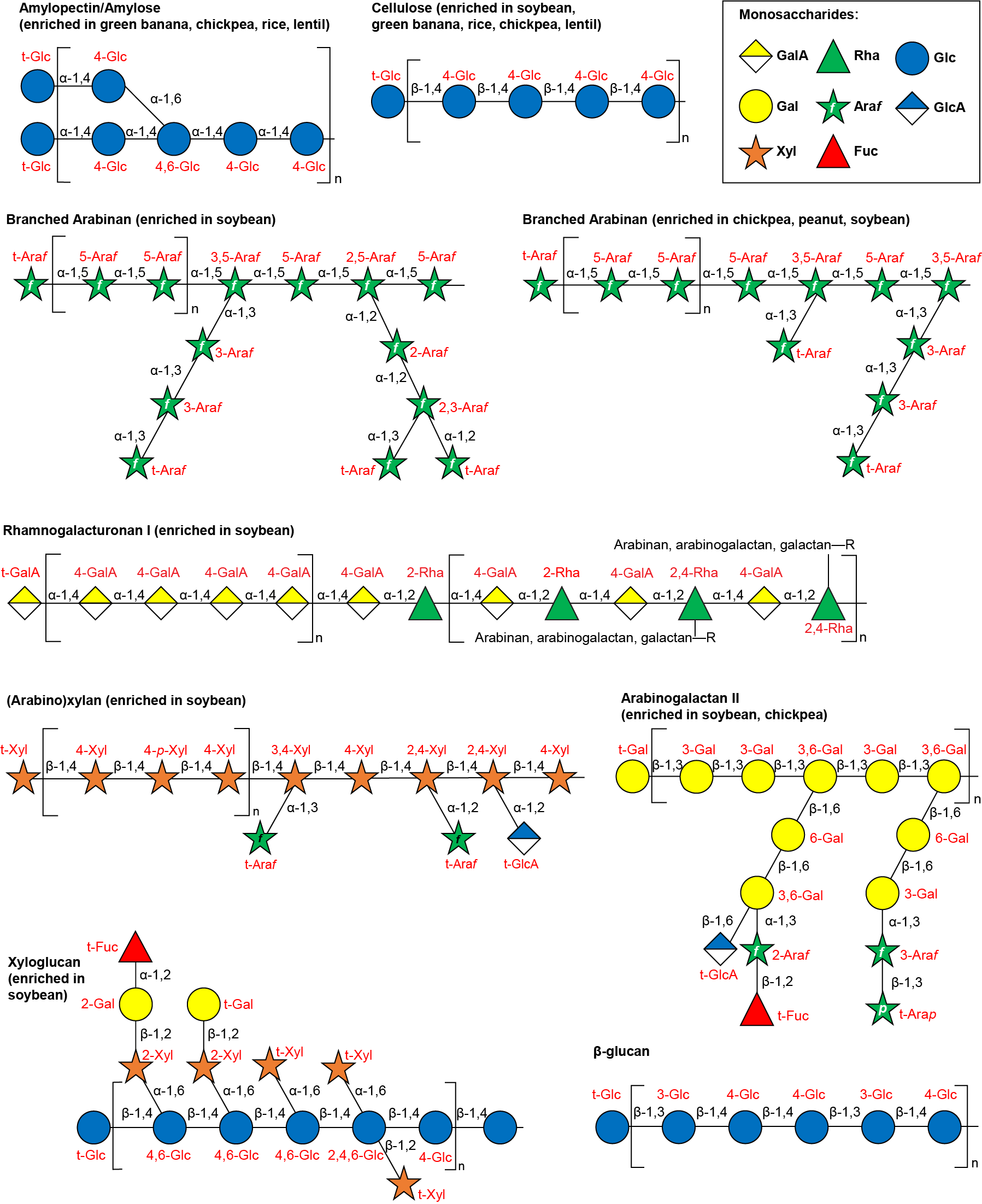


Supplementary Fig. 2. Structures of glycans enriched in components of MDCF-2 or RUSF.


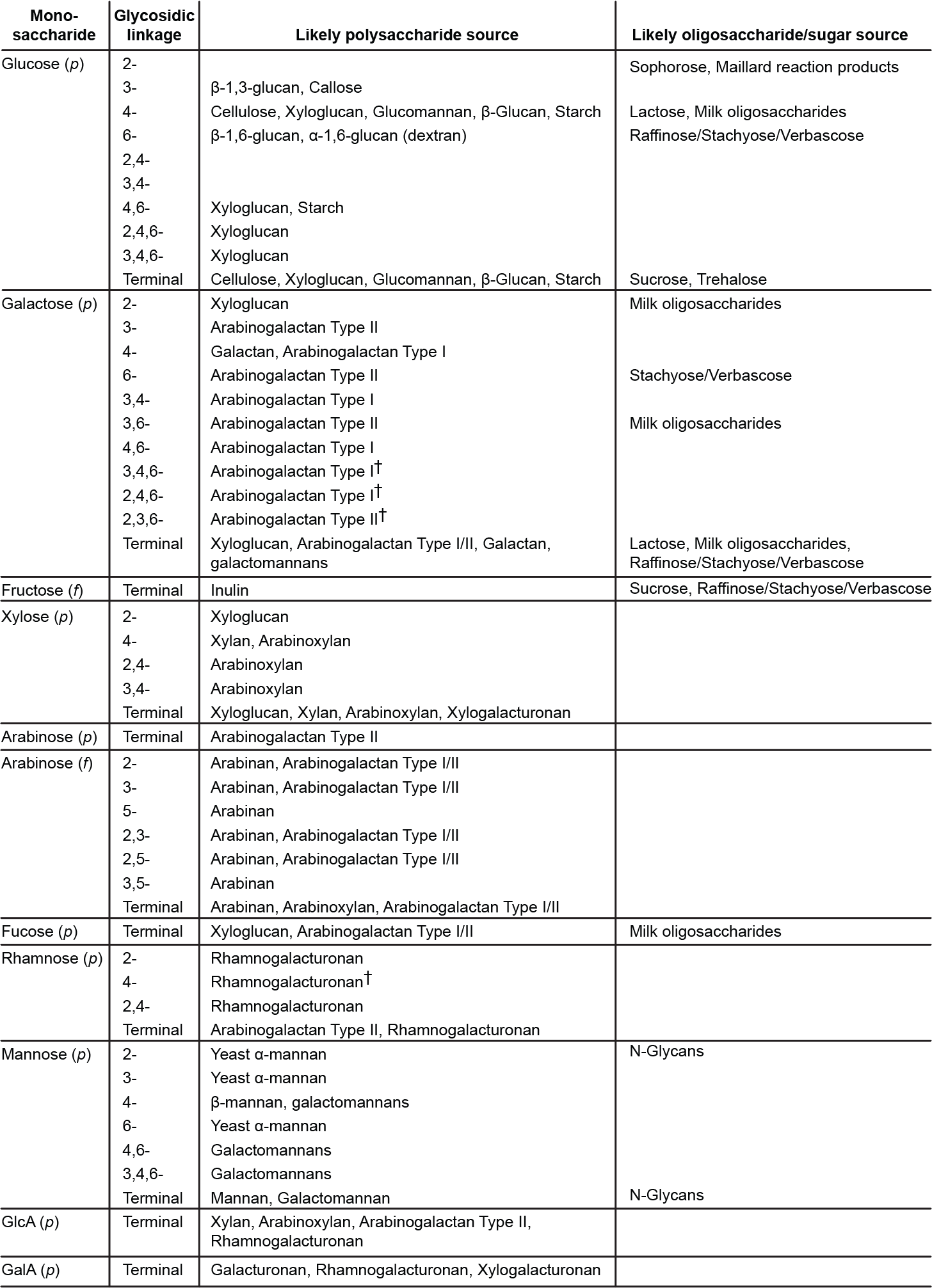
Supplementary Fig. 3. LC-MS analysis of glycans present in MDCF-2, RUSF and their component ingredients. Measured glycosidic linkages in MDCF-2 and RUSF and their likely polysaccharide sources. Sources were inferred based on analysis of literature as well as polysaccharide standards. ‘†’ refers to instances where literature reports were not available and commercial polysaccharide preparations were used for direct experimental validation.


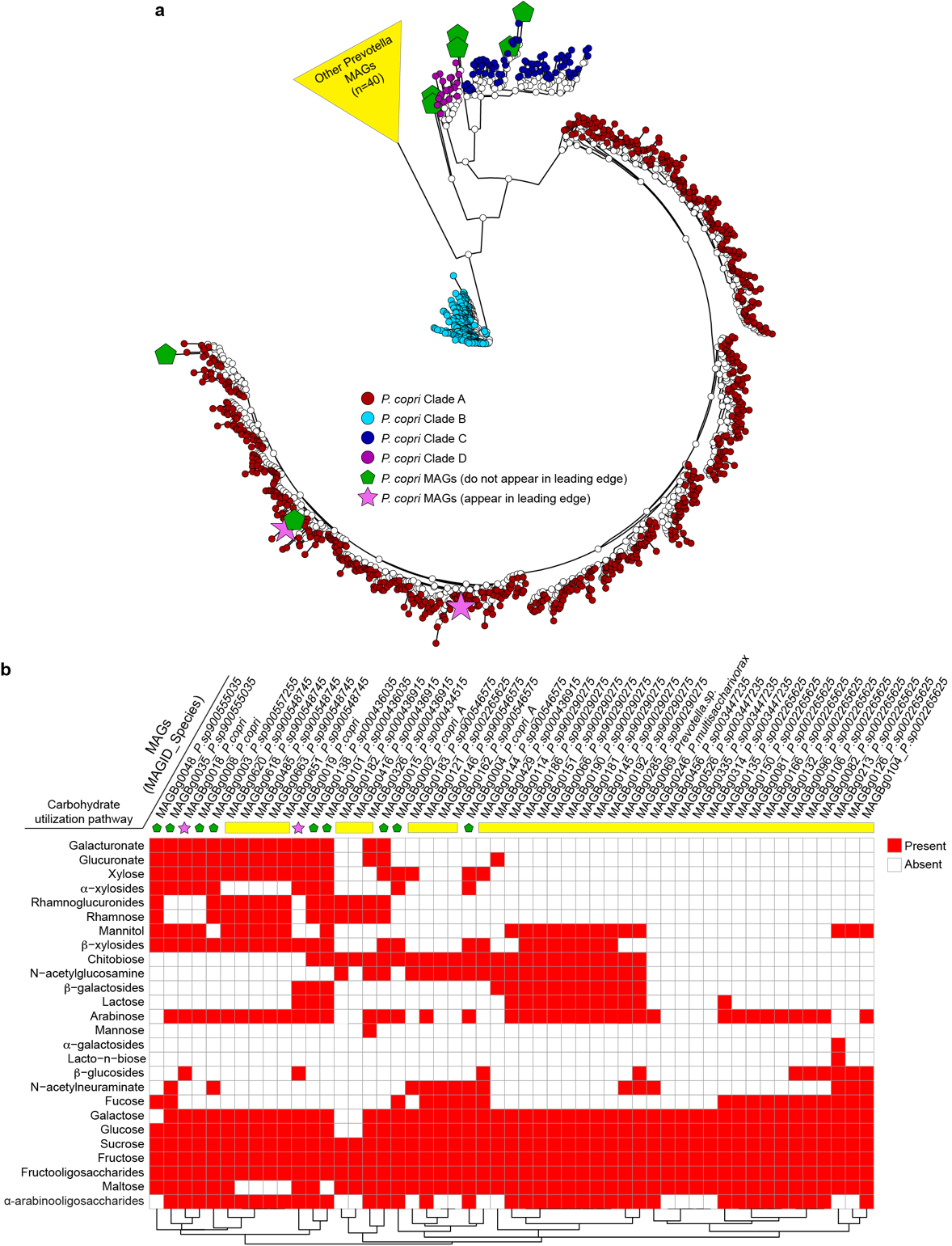


Supplementary Fig. 4. Phylogenetic tree and representation of mcSEED carbohydrate utilization pathways in *Prevotella* MAGs (a) Unrooted, marker gene-based phylogenetic tree of 51 *Prevotella* MAGs from this study, plus 1,049 *P. copri* genomes and MAGs previously assigned to each of four clades^19^. Pink stars denote the two WLZ-associated *P. copri* MAGs. The nine remaining *P. copri* MAGs from this study are highlighted by the green pentagons. The 40 *Prevotella* MAGs not classified as *P. copri*, based on an average branch length >0.5 from all 1,049 reference *P. copri* isolates, are grouped together and depicted as a yellow triangle. (b) mcSEED carbohydrate utilization pathways in 51 *Prevotella* MAGs from the current study. MAGs are hierarchically clustered based on the predicted presence (red) or absence (white) of these pathways.

**
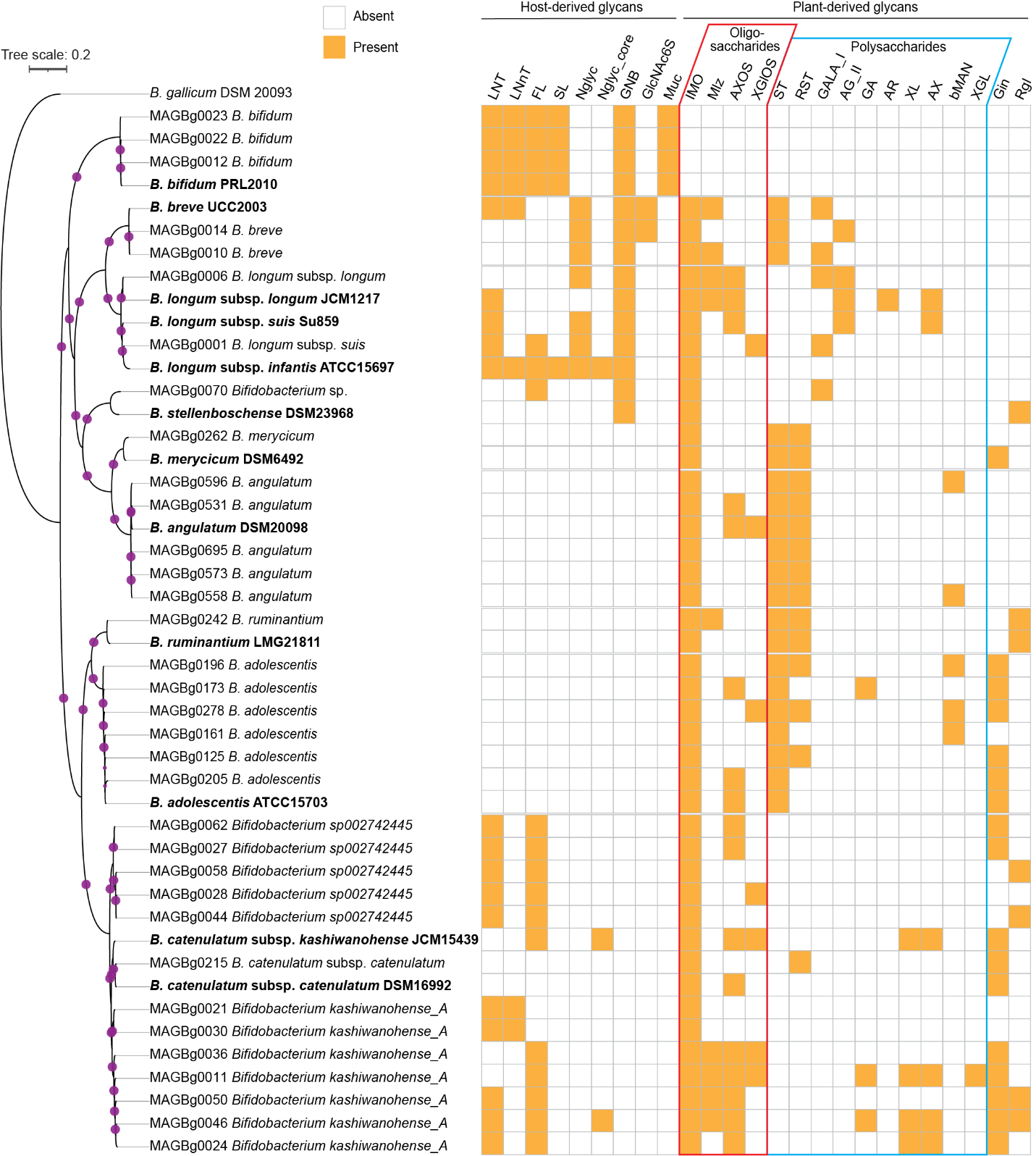
**

**Supplementary Fig. 5.** **Phylogenetic tree and inferred carbohydrate utilization phenotypes of *Bifidobacterium* MAGs.** The phylogenetic tree indicates the relatedness of 34 *Bifidobacterium* MAGs and 14 reference genomes, as determined by sequence similarity among 142 core genes. The size of the pink circles in the dendrogram correspond to bootstrap support for the nodes (out of 100 bootstraps). Type strains used for taxonomic assignments and phenotypic comparisons are bolded. The matrix describes the presence (orange) or absence (white) of 25 predicted carbohydrate utilization phenotypes encompassing host- and plant-derived glycans. LNT, lacto-*N*-tetraose; LNnT, lacto-*N*-neotetraose; FL, 2- and 3-fucosyllactose; SL, 3- and 6-sialyllactose; Nglyc, *N*-glycans; Nglyc_core, *N*-glycan core (Fucα1-6GlcNAcβ1-Asn); GNB, galacto-*N*-biose; GlcNAc6S, *N*-acetylglucosamine-6-sulfate; Muc, mucin *O*-glycans; IMO, isomaltooligosaccharides and panose; Mlz, melezitose; AXOS, arabinoxylooligosaccharides; XGlOS, xyloglucan oligosaccharides; ST, starch and glycogen; RST, resistant starch; GALA_I, type I galactan and arabinogalactan; AGII, type II galactan and arabinogalactan; GA, gum arabic; AR, arabinan; XL, xylan; AX, arabinoxylan; bMAN, β-mannan; XGL, xyloglucan; Gin, ginsenosides; Rgl, rhamnoglycosides.
