# Supplementary material for: Bioactive glycans in a microbiome-directed food for children with malnutrition
Source: Nature. 2023 Dec 13;625(7993):157–65. doi: 10.1038/s41586-023-06838-3 (PMC10764277; doi:10.1038/s41586-023-06838-3)
Supplement: Supplementary file 2 — Reporting Summary [file 41586_2023_6838_MOESM2_ESM.pdf]

nature portfolio

Corresponding author(s): Jeffrey Gordon

Last updated by author(s): Oct 31, 2023

Reporting Summary

Nature Portfolio wishes to improve the reproducibility of the work that we publish. This form provides structure for consistency and transparency in reporting. For further information on Nature Portfolio policies, see our Editorial Policies and the Editorial Policy Checklist.

Statistics

For all statistical analyses, confirm that the following items are present in the figure legend, table legend, main text, or Methods section.

n/a	Confirmed	
☐	☒	The exact sample size (*n*) for each experimental group/condition, given as a discrete number and unit of measurement
☐	☒	A statement on whether measurements were taken from distinct samples or whether the same sample was measured repeatedly
☐	☒	The statistical test(s) used AND whether they are one- or two-sided<br>*Only common tests should be described solely by name; describe more complex techniques in the Methods section.*
☐	☒	A description of all covariates tested
☐	☒	A description of any assumptions or corrections, such as tests of normality and adjustment for multiple comparisons
☐	☒	A full description of the statistical parameters including central tendency (e.g. means) or other basic estimates (e.g. regression coefficient) AND variation (e.g. standard deviation) or associated estimates of uncertainty (e.g. confidence intervals)
☐	☒	For null hypothesis testing, the test statistic (e.g. *F*, *t*, *r*) with confidence intervals, effect sizes, degrees of freedom and *P* value noted<br>*Give P values as exact values whenever suitable.*
☐	☒	For Bayesian analysis, information on the choice of priors and Markov chain Monte Carlo settings
☐	☒	For hierarchical and complex designs, identification of the appropriate level for tests and full reporting of outcomes
☐	☒	Estimates of effect sizes (e.g. Cohen's *d*, Pearson's *r*), indicating how they were calculated

Our web collection on statistics for biologists contains articles on many of the points above.

Software and code

Policy information about availability of computer code

Data collection	No specialized software or code was used for data collection
Data analysis	The following software packages were used to analyze the data in this study: Trim Galore3 (v0.6.4), bowtie2 (v2.3.4.1), MegaHit (v1.1.4), MaxBin2 (v2.2.5), MetaBAT2 (v2.12.1), DAS Tool (v1.1.2), OPERA-MS (v0.9.0), CheckM (v1.1.3), MAGpurify (v2.1.2), dRep (v2.6.2), prokka (v1.14.6), GTDB-Tk (v1.3.0), Kraken2 (v2.0.8), Bracken (v2.5), Phylophlan (v3.0.60), Graphlan (v1.1.4), Roary (v3.12.0), MAFFT (v7.313), microseq R package (v2.1.4), IQ-TREE (v1.6.12), kallisto (v0.43.0), DESeq2 (v1.34.0), lme4 (v1.1-27.1), lmerTest (v3.1-3), dream variancePartition R package (v1.24.0), FastQC (v0.11.7), edgeR37 (v3.32.1), factoextra (v1.0.7), vegan R package (v2.5-7), ggtree (v3.2.1), ape (v5.6-2), MMSeqs (v1-c7a89), DIAMOND (v2.0.0), prodigal (v2.6.3), Scikit-learn (v0.22.1), Rpart (v4.1.15), Caret (v6.0.86). Code detailing the steps in the MAG assembly workflow and analyses of microbial RNA-Seq and glycan datasets are available from GitLab (https://gitlab.com/hibberdm/hibberd_webber_et_al_mdcf_poc_mags) and have been accessioned at Zenodo (DOI:10.5281/zenodo.8000098). Code for annotation of bacterial genes and prediction of metabolic phenotypes is available from GitHub (https://github.com/rodionovdima/PhenotypePredictor) and has been accessioned at Zenodo (DOI: 10.5281/zenodo.10049439).

For manuscripts utilizing custom algorithms or software that are central to the research but not yet described in published literature, software must be made available to editors and reviewers. We strongly encourage code deposition in a community repository (e.g. GitHub). See the Nature Portfolio guidelines for submitting code & software for further information.

Data

Policy information about availability of data

All manuscripts must include a data availability statement. This statement should provide the following information, where applicable:

- Accession codes, unique identifiers, or web links for publicly available datasets
- A description of any restrictions on data availability
- For clinical datasets or third party data, please ensure that the statement adheres to our policy

Shotgun DNA sequencing and microbial RNA-Seq datasets generated from fecal samples, plus annotated P. copri isolate genome sequences are available in the European Nucleotide Archive (accession PRJEB45356). Anthropometry data is available from a prior publication (ref. 4; DOI: 10.1056/NEJMoa2023294). The mcSEED database (https://zenodo.org/records/10041396) was used to predict the presence or absence of metabolic pathways. The Genome Taxonomy Database (GTDB) was used as a reference for taxonomic classification of MAGs. The CAZy and PULdb databases were used to identify and analyze carbohydrate-active genes and PULs. LC-MS datasets of monosaccharide, glycoside linkage, and polysaccharide data are deposited in GlycoPOST (accession GPST000244). All other relevant and/or supporting data is available in the Supplementary Information.

Field-specific reporting

Please select the one below that is the best fit for your research. If you are not sure, read the appropriate sections before making your selection.

☒ Life sciences ☐ Behavioural & social sciences ☐ Ecological, evolutionary & environmental sciences

For a reference copy of the document with all sections, see nature.com/documents/nr-reporting-summary-flat.pdf

Life sciences study design

All studies must disclose on these points even when the disclosure is negative.

Sample size	Target enrollment for the clinical trial was set at 124 participants (n=62 per arm). This sample size was calculated to achieve 80% power at a 5% significance level for detecting a treatment effect. The anticipated effect size was derived from changes in Weight-for-Length Z-score (WLZ) observed in a pilot randomized, double-blind controlled feeding study (ref. 3, DOI: 10.1126/science.aau4732). A total of 123 participants were randomized in the clinical trial (n=61 MDCF-2 diet, 62 RUSF diet), and 59 children in each group completed the 3-month intervention and 1-month follow-up and were included in our analyses. Additional details on the clinical trial design and sample size calculations are available elsewhere (ref. 4 [DOI: 10.1056/NEJMoa2023294], DOI 10.1186/s12889-020-8330-8).
Data exclusions	Participants who did not complete the trial were excluded as described previously (ref. 4 [DOI: 10.1056/NEJMoa2023294]). No additional data were excluded from the analyses described in the current study.
Replication	To enhance reproducibility, the MAG assembly pipeline was validated using synthetic sequence data from CAMI II as indicated in Extended Data Fig. 10. This validation measured the fidelity of MAG assembly, the accuracy of kallisto-based MAG quantification, and the effects of filtering as described in the supplementary information. We employed a consensus-based approach for assigning annotations as a means of enhancing the reliability of these metabolic pathway annotations. We utilized both short- and long-read MAG assembly methods and compared these assembly methods as illustrated in Extended Data Fig. 10. We compared results from two methods of taxonomic assignment and utilized a consensus approach to assign MAG taxonomy in a reproducible manner. The capacity of P. copri strains to utilize specific carbohydrate substrates was validated using a series of in vitro growth experiments as illustrated in Extended Data Fig. 5.
Randomization	Eligible participants, whose parents/guardians provided written informed consent, were randomly assigned to treatment groups. Participant randomization was performed by an independent researcher who has had no involvement in the trial (please see an additional description of randomization: DOI 10.1186/s12889-020-8330-8).
Blinding	Participants were blinded to their treatment group. Study staff were blinded to the extent that was possible as described in DOI 10.1186/s12889-020-8330-8. Investigators were not blinded to group allocation when analyzing the data presented in this paper.

Reporting for specific materials, systems and methods

We require information from authors about some types of materials, experimental systems and methods used in many studies. Here, indicate whether each material, system or method listed is relevant to your study. If you are not sure if a list item applies to your research, read the appropriate section before selecting a response.

Materials & experimental systems

n/a	Involved in the study	
☒	☐	Antibodies
☒	☐	Eukaryotic cell lines
☒	☐	Palaeontology and archaeology
☒	☐	Animals and other organisms
☐	☒	Human research participants
☐	☒	Clinical data
☒	☐	Dual use research of concern

Methods

n/a	Involved in the study	
☒	☐	ChIP-seq
☒	☐	Flow cytometry
☒	☐	MRI-based neuroimaging

Human research participants

Policy information about studies involving human research participants

Population characteristics	Please refer to Chen, Mostafa, Hibberd et al., "A Microbiota-Directed Food Intervention for Undernourished Children", N Engl J Med 2021 (DOI: 10.1056/NEJMoa2023294) for population characteristics.
Recruitment	Please refer to Chen, Mostafa, Hibberd et al., "A Microbiota-Directed Food Intervention for Undernourished Children", N Engl J Med 2021 (DOI: 10.1056/NEJMoa2023294) for recruitment information.
Ethics oversight	The study protocol was approved by the Ethical Review Committee at the International Center for Diarrheal Disease Research, Bangladesh (icddr,b)

Note that full information on the approval of the study protocol must also be provided in the manuscript.

Clinical data

Policy information about clinical studies

All manuscripts should comply with the ICMJE guidelines for publication of clinical research and a completed CONSORT checklist must be included with all submissions.

Clinical trial registration	ClinicalTrials.gov identifier: NCT04015999
Study protocol	The human biospecimens analyzed in this manuscript are from a previously reported clinical study. The protocol for this study is detailed in ref. 4 (N Engl J Med 2021 (DOI: 10.1056/NEJMoa2023294).
Data collection	The human biospecimens analyzed in this manuscript are from a previously reported clinical study. The data collection methods for this study are detailed in ref. 4 (N Engl J Med 2021 (DOI: 10.1056/NEJMoa2023294).
Outcomes	The human biospecimens analyzed in this manuscript are from a previously reported clinical study. The primary and secondary outcome measures for this study are detailed in ref. 4 (N Engl J Med 2021 (DOI: 10.1056/NEJMoa2023294).
